# Role of the type VI secretion systems during disease interactions of *Erwinia amylovora* with its plant host

**DOI:** 10.1186/s12864-017-4010-1

**Published:** 2017-08-17

**Authors:** Tim Kamber, Joël F. Pothier, Cosima Pelludat, Fabio Rezzonico, Brion Duffy, Theo H. M. Smits

**Affiliations:** 1 Agroscope Changins-Wädenswil ACW, Plant Protection Division, 8820 Wädenswil, CH Switzerland; 2 Environmental Genomics and Systems Biology Research Group, Institute of Natural Resource Sciences, Zurich University of Applied Sciences (ZHAW), 8820 Wädenswil, CH Switzerland

**Keywords:** Fire blight, RNA-sequencing, Motility, Flagella

## Abstract

**Background:**

Type VI secretion systems (T6SS) are widespread among Gram-negative bacteria and have a potential role as essential virulence factors or to maintain symbiotic interactions. Three T6SS gene clusters were identified in the genome of *E*. *amylovora* CFBP 1430, of which T6SS-1 and T6SS-3 represent complete T6SS machineries, while T6SS-2 is reduced in its gene content.

**Results:**

To assess the contribution of T6SSs to virulence and potential transcriptomic changes of *E. amylovora* CFBP 1430, single and double mutants in two structural genes were generated for T6SS-1 and T6SS-3. Plant assays showed that mutants in T6SS-3 were slightly more virulent in apple shoots while inducing less disease symptoms on apple flowers, indicating that T6SSs have only a minor effect on virulence of *E*. *amylovora* CFBP 1430. The mutations led under in vitro conditions to the differential expression of type III secretion systems, iron acquisition, chemotaxis, flagellar, and fimbrial genes. Comparison of the *in planta* and in vitro transcriptome data sets revealed a common differential expression of three processes and a set of chemotaxis and motility genes. Additional experiments proved that T6SS mutants are impaired in their motility.

**Conclusion:**

These results suggest that the deletion of T6SSs alters metabolic and motility processes. Nevertheless, the difference in lesion development in apple shoots and flower necrosis of T6SS mutants was indicative that T6SSs influences the disease progression and the establishment of the pathogen on host plants.

**Electronic supplementary material:**

The online version of this article (doi:10.1186/s12864-017-4010-1) contains supplementary material, which is available to authorized users.

## Background

Type VI secretion systems (T6SS) were discovered in ecologically-diverse pathogenic and non-pathogenic Gram-negative bacteria, including bacteria associated with eukaryotic cells maintaining pathogenic or symbiotic interactions [1–3]. T6SS gene clusters characteristically consist of a set of 13 core genes and a varying number of accessory genes [4] including Hcp and VgrG that were frequently identified in culture supernatants [5–7]. In some bacterial species Hcp and VgrG along with additional effectors were discovered to have antibacterial properties that are targeted to recipient cells and contribute to the competitiveness and indirectly to virulence [8–13]. The T6SS are implied in various functions in different bacterial species, e.g., biofilm formation, inter-bacterial pathogenicity, host-cell invasion and survival within macrophages [8, 14–19]. The contribution of T6SS to virulence was demonstrated for certain animal and human pathogens [20–24], whereas only few reports about functions in plant associated bacteria exist [25–29]. In *Rhizobium leguminosarum,* the T6SS was described as a nodulation impairment locus *imp* [30] and later identified as a secretion system [31]. First indications that T6SS might contribute to virulence in plant pathogenic bacteria came from microarray profiling and secretome analysis in *Pectobacterium atrosepticum* SCRI1043 and *Agrobacterium tumefaciens* C58 [6, 7, 28]. In *A*. *tumefaciens* C58, it was shown that the T6SS is involved in virulence and interbacterial competition [7, 8]. A contribution to virulence was also demonstrated for *Ralstonia solanacearum* where mutation of a T6SS gene led to an attenuation of virulence on tomato plants and additionally to a reduction of motility and biofilm formation [25]. The mutational analysis of the T6SSs in *Pantoea ananatis* showed that one of the clusters is essential for pathogenicity on onion host plants and is involved in bacterial competition [26].

*Erwinia amylovora* is a Gram-negative, enterobacterial phytopathogen causing the fire blight disease [32]. The pathogen has a broad host-range affecting various *Rosaceae* (primarily Spiraeoideae), including ecologically and economically important species (e.g., apple and pear). The pathogenicity of *E*. *amylovora* is strictly dependent on a functional type III secretion system (T3SS) and the production of the exopolysaccharide amylovoran [33]. We identified three T6SS (T6SS-1-T6SS-3) in *E. amylovora* CFBP 1430, of which two include the 13 core genes, whereas the third cluster is only rudimentary [34]. T6SS-1 and T6SS-2 were also identified in other *Erwinia* species, whereas the third cluster is not present in all of them [35]. Recently, it was demonstrated that the T6SSs of *E. amylovora* are involved in bacterial competition, exopolysaccharide production, and virulence on immature pear fruits [36]. Bacterial competition was influenced by all T6SS mutants tested, whereas the effects on exopolysaccharide production and virulence varied between the different gene knock-out variants [36]. In this study, the aim was to analyze a potential contribution of the T6SSs of *E*. *amylovora* in virulence to plants, antibacterial properties, and to use RNA-seq to analyze transcriptomic changes. The performed plant assays indicated a minor contribution of T6SSs to the virulence *E*. *amylovora* CFBP 1430. Additionally, competition assays showed that deletion of the T6SSs slightly affected the antibacterial properties of *E*. *amylovora* CFBP 1430. Differential expression of motility and chemotaxis genes were observed, and further experiments showed that the T6SSs have an effect on the motility of *E*. *amylovora* CFBP 1430.

## Methods

### Bacterial strains, media, and growth conditions

Bacterial strains used in this study (Table 1) were routinely grown on KB [37] or LB (Roth, Karlsruhe, Germany) plates with the addition, where necessary, of chloramphenicol (20 μg ml^−1^), nalidixin (35 μg ml^−1^) and ampicillin (200 μg ml^−1^). For flower population studies, NSA plates (8 g l^−1^ nutrient broth, 50 g l^−1^ sucrose) containing actidione (50 mg l^−1^) were used. Liquid cultures were grown to the mid-log phase at 28 °C with continuous shaking. Cells for RNA extraction were either grown in M9 sucrose (Difco), LB, KB, M63 glucose (13.6 g l^−1^ KH_2_PO_4_, 2.0 g l^−1^ (NH_4_)_2_SO_4_, 0.005 g l^−1^ FeSO_4_·7H_2_O, 0.246 g l^−1^ MgSO_4_·7H_2_O, and 0.4 g l^−1^ glucose) or HrpMM supplemented with sorbitol or sucrose (0.55 g l^−1^ KH_2_PO_4_, 0.15 g l^−1^ K_2_HPO_4_, 0.1 g l^−1^ (NH_4_)_2_SO_4_, 0.0344 g l^−1^ MgCl_2_, 0.01 g l^−1^ NaCl, and 0.4 g l^−1^ carbohydrate source).

**Table 1:** Bacterial strains and plasmids used in this study

Strain or plasmid	Description	Reference
*E*. *amylovora* CFBP 1430	Wild type strain	[68]
*E*. *amylovora* T6-d1	Δ*tssB-1*/*tssC-1*T6SS cluster 1 deletion mutant, Cm^R^	This study
*E*. *amylovora* T6-d3	Δ*tssB-2*/*tssC-2* T6SS cluster 3 deletion mutant, Cm^R^	This study
*E*. *amylovora* T6-d1d3	Δ*tssB*/*tssC* T6SS cluster 1 and 3 deletion mutant, Cm^R^	This study
*E. amylovora* Δ*hrpL*	Δ*hrpL* Deletion mutant, Cm^R^	[44]
*E. coli* DH5α	Φ*80 lacZ M15*, Δ(*lacZYA-argF*) *U169*, *recA1*, *endA1,thi-1*	Lab strain
pKD46	Red recombinase expressing plasmid, Ap^R^	[38]
pKD3	Antibiotic resistance cassette template, Ap^R^, Cm^R^	[38]
pCP20	FLP encoding recombinase plasmid, Ap^R^, Cm^R^	[38]

*Ap* ^*R*^ ampicillin resistance, *Cm* ^*R*^ chloramphenicol resistance

### Construction of T6SS mutants

The lambda red recombination system [38] was used to replace consecutive genes EAMY_3020–3021 and EAMY_3227–3228 (coding for TssB1/TssC1 and TssB2/TssC2) in both T6SS clusters. The resulting single mutants were named T6-d1, T6-d3, and the double mutant T6-d1d3. Plasmids used are listed in Table 1. Plasmid pKD3 was used as template to amplify the chloramphenicol resistance cassette with the primer pairs listed in Additional file 1: Table S1. PCR products were introduced by electroporation into competent *E. amylovora* CFBP 1430 cells carrying plasmid pKD46. The single mutants were constructed by deletion of the complete reading frames of *tssB*/*tssC* in each cluster. To create the double mutant, the plasmid pKD46 was removed from the T6-d1 by overnight incubation on LB plates at 37 °C and colonies were screened for loss of antibiotic resistance. Plasmid pCP20 was introduced to eliminate the chloramphenicol resistance cassette and mutation of *tssB*/*tssC* in T6SS-3 was performed as described above. All knock-out mutants were confirmed by PCR and sequencing.

### Immature pear assay

*E*. *amylovora* WT, T6-d1, T6-d3, T6-d1d3, and a Δ*hrpL* mutant were grown overnight in KB liquid cultures, collected by centrifugation, adjusted to an OD_600_ = 0.1 (i.e., approximately 10^8^ cfu ml^−1^) and diluted to cell-densities of approximately 10^3^, 10^5^, and 10^7^ cfu ml^−1^. The pears (cultivar ‘Conférence’) were surface sterilized using 10% NaOCl, pierced with a needle and subsequently inoculated with 5 μl bacterial suspension or as a control with water [39, 40]. The fruits were incubated in a humidity chamber at 28 °C for 8 days. Fruits were assayed in triplicates for each treatment and the experiment was repeated twice.

### Shoot inoculation assays

Shoots of potted one-year old grafted apple (*Malus* × *domestica* cv. ‘Golden Delicious’) and pear plants (*Pyrus communis* cv. ‘Conférence’) were used for inoculation experiments. The plants were kept at 20 °C during the day and 18 °C at night with a relative humidity of 80%. Shoots were inoculated using a syringe with a bacterial suspension containing approximately 10^8^ cfu ml^−1^. The plants were assayed in pentaplicates with five repetitions. The disease progress was recorded weekly over four weeks and calculated as the percentage of total shoot length with observable lesion formation [39].

### Flower assay

Newly opened flowers of two-year-old grafted apple (*Malus* × *domestica* cv. ‘Golden Delicious’) trees were spray-inoculated (200 μl/spray dose) using an atomizer. The bacterial suspensions contained approximately 10^7^ cfu ml^−1^. To determine bacterial population sizes, the petals were removed and the remaining flower parts were washed in water. Bacteria were plated immediately after inoculation, 1 DPI (day post inoculation) and 2 DPI post inoculation. Twenty flowers per tree were assessed to determine the population sizes. Flower necrosis was rated at 10 DPI and 12 DPI. The experiment was repeated twice. A total of 529 (WT), 629 (T6-d1), 543 (T6-d3) and 580 (T6-d1d3) flowers were rated.

### RNA isolation

RNA from bacteria grown to the mid-log phase in liquid media was extracted using the NucleoSpin RNA II kit (Macherey Nagel, Oensingen, Switzerland) following manufacturer instructions. Flowers were inoculated with a pipette applying to the hypanthium 5 μl of bacterial suspension containing 10^7^ cfu ml^−1^. The flowers were collected 2 DPI, petals were removed and washed in water. The floral parts were removed from the solution and centrifuged. The supernatant was discarded, the pellet flash-frozen in liquid nitrogen and stored at −86 °C until RNA-extraction. Total RNA was isolated using the innuPREP Plant RNA Kit (Analytikjena, Jena, Germany) according to the manufacturer’s instructions. All total RNA samples were treated with DNase I (Thermo Scientific, Wohlen, Switzerland) and control PCRs were performed to check for absence of DNA contamination. Quality and concentration of the RNA samples were determined using the Bioanalyzer 2100 (Agilent Technologies, Stuttgart, Germany). Samples were pooled to the required amount for further processing.

### Reverse-transcription RT-PCR

The RevertAid H Minus First Strand cDNA Synthesis Kit (Fermentas, Reinach, Switzerland) was used to create cDNA templates for RT-PCR. Aliquots of 1 μg of RNA were reversed transcribed with random hexamers according to manufacturer instructions and used for PCR with the primer pairs listed in Additional file 1: Table S1.

### RNA-seq data analysis

The cDNA libraries, derived from liquid cultures (ribosomal RNA depleted), were constructed and Illumina sequenced leading to 18,598,523 (WT) and 18,275,977 (T6-d1d3) reads. The reads were aligned with Bowtie version 0.12.7 [41] to the *E*. *amylovora* CFBP 1430 genome sequence [34] with an overall alignment rate of 91.43% (WT) and 91.04% (T6-d1d3). The cDNA libraries, derived from inoculated flowers, led to 44,296,072 (WT inoculated sample) and 45,470,859 (T6-d1d3 inoculated sample) reads. Alignment of the reads to the *E. amylovora* CFBP 1430 genome sequence had overall alignment rate of 10.65% (WT inoculated sample) and 20.77% (T6-1d-3 inoculated sample). Analysis of differential expression levels was performed using Cufflinks version 1.3.0 [42]. Gene expression levels were normalized using fragments per kb of exon per million mapped reads (FPKM) report values. Genes were considered as significantly differentially expressed, when their fold change was ≥1.5 or ≤ −1.5, respectively, and their *p* value <0.001.

### Motility assay

WT and T6SS mutants were freshly plated from glycerol stocks kept at −86 °C and grown overnight on KB plates. The bacteria were collected and resuspended in 0.8% NaCl, washed three times with 0.8% NaCl by centrifugation. The bacterial strains were adjusted to an OD_600_ = 0.1 (10^8^ cfu ml^−1^) and 20 μl of the suspension was spotted in the middle of soft agar plates (3 g l^−1^ agar, 10 g l^−1^ plant tryptone, 5 g l^−1^ NaCl). The motility agar plates were assessed after 2 days incubation at 28 °C.

### Competition assay

All strains were grown overnight in LB, washed once with 0.8% NaCl and adjusted to OD_600_ = 0.900. The WT or one of the T6SS mutants was mixed in a ratio of 1:1 with *Escherichia coli* DH5α cells and 20 μl of suspension was spotted on LB and KB agar plates. The spots were excised after 24 h from the agar and resuspended in 2 ml NaCl, vortexed, and serial dilution was performed. The plates were incubated at 30 °C overnight and subsequently cfu were counted.

### Statistics

Statistical analyses were performed with the Sigmaplot software Version 10 (Systat Software, San Jose, USA) or using R version 3.2.3. The lesion length data were log transformed prior to analysis as a two-way ANOVA with mean comparisons evaluated using Fisher’s least significant difference test at a significance level of 5%. The flower necrosis data were assessed with a one-way ANOVA using the Fisher’s LSD test at a significance level of 5% to test for statistical significant differences between treatments. The competition assays were analyzed using a one-way ANOVA with a Tukey posthoc test.

## Results

### Generation and characterization of T6SS mutants

**Fig. 1:**
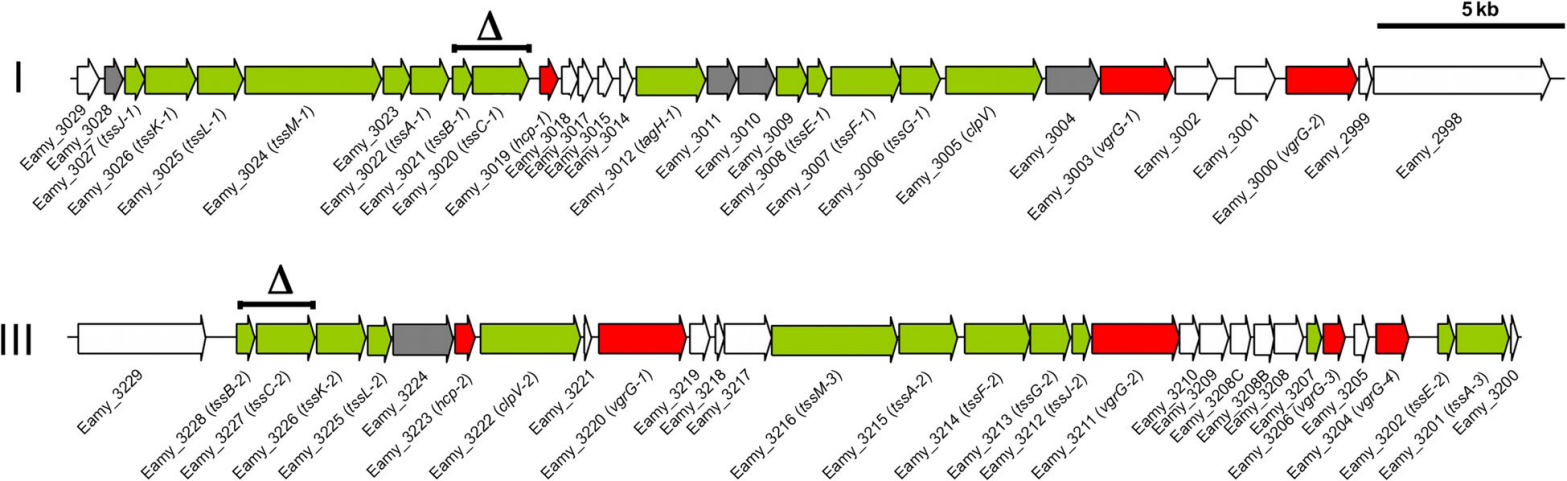
Type VI secretion systems gene clusters of *Erwinia amylovora* CFBP 1430. Core genes are depicted in *green*, putative effectors in *red*, conserved genes found in homologous clusters in *grey*. Deleted genes in either T6SS cluster 1 and/or cluster 3 are marked with *a Δ* and *a bar*

*E*. *amylovora* CFBP 1430 has three T6SS clusters (T6SS-1-T6SS-3) that differ in core and accessory gene content as well as gene organization [34]. T6SS-2 (EAMY_1620–1623) is constituted of four genes and hence not a complete T6SS gene cluster. Therefore we focused on T6SS-1 and T6SS-3 (EAMY _3003–3028 and EAMY_3201–3228) (Fig. 1). Both T6SS gene clusters possess one *hcp* and two *vgrG* genes, with no evolved effector domains [35]. Genes coding for potential signal transducers (serine/threonine phosphatase and serine/threonine kinase) are present in T6SS-1, but absent in T6SS-3.

The T6SS gene clusters have been described, whereas their activity and role in pathogenicity were not. Therefore, to test whether T6SS related genes were transcribed under different conditions, we analyzed the expression of a set of selected T6SS genes (T6SS-1 (*hcp-1*, *tssF-1*), T6SS-3 (*hcp-2*, *vgrG-4*, *tssF-2*)), by isolating RNA from *E. amylovora* CFBP 1430 grown in different liquid media. The qualitative assessment by RT-PCR (Additional file 1: Figure S1) showed that the T6SSs genes in *E*. *amylovora* CFBP 1430 were expressed under the tested conditions. This indicated that these genes represent an active secretion system, which can be assessed both in vitro and in vivo.

Deletion mutants were constructed in order to determine whether the T6SS-1 and T6SS-3 contribute to the virulence of *E*. *amylovora* CFBP 1430. To exclude that the mutations have an influence on the growth rates of the mutants, growth curves were determined in different liquid media. No obvious differences in the growth rates of the individual mutants compared to the wildtype strain were detected (Additional file 1: Figure S2), which allows the direct comparison of the strains both in vitro and in vivo.

### In vitro transcriptional differences between *E. amylovora* CFBP 1430 and the T6SS double mutant T6-d1d3

The cDNA libraries for Illumina sequencing were constructed from RNA isolated from WT and T6-d1d3 mutant strains of *E*. *amylovora* CFBP 1430, grown in minimal M9 medium supplemented with sucrose. The comparison of the WT to the T6-d1d3 mutant revealed differential expression of 508 genes (Additional file 1: Table S2): of these, 391 genes have an annotation and 117 are predicted genes. The differential expressed genes were classified into groups according to their gene ontology (GO) terms (Additional file 1: Figure S3). Processes affected include the categories cellular process, metabolic process, pigmentation, stimuli response, biological regulation, localization and locomotion.

Nine genes of the hypersensitive response and pathogenicity (Hrp) cluster (Table 2), the putative effector *hopPtoC*, and genes of the two Inv/Spa-type III secretion systems (PAI-2, PAI-3) were differentially expressed (Table 2). The transcripts of the Hrp gene cluster had lower transcript abundance in the T6-d1d3 mutant than transcripts of the WT with the exception of the putative translocator *hrpK* [43], which showed higher abundance. Genes of the PAI-2 Inv/Spa cluster had higher transcript abundance in T6-d1d3 than in the WT, whereas transcripts of the PAI-3 Inv/Spa were less abundant, except for the putative chaperone gene *spaK,* which was higher. Genes for amylovoran (*amsK, amsL, amsJ*) and desferrioxamine siderophore biosynthesis (*dfoJ*) were also influenced by T6SS mutation. The transcripts levels of *amsK* and *amsL* were lower while *amsJ* was higher in the T6-d1d3 mutant compared to the WT. Transcripts of the two sets of flagellar genes were differentially expressed, most biosynthetic and associated chemotaxis and motility (*fli*, *flh* and *flg* and all *mot* and *che*) genes had a higher transcript abundance in the T6-d1d3 mutant than in the WT, except the biosynthesis genes *fliQ3*, *fliR3* and transcriptional regulators *flhD3* and *flgM* which were lower.

**Table 2:** Summary table of selected significantly differentially expressed genes from the in vitro experiment comparing the WT to the T6SS double mutant

Name	Description	Locus tags	*p* value	Fold change
Chemotaxis and motility				
*cheW3* *cheA3* *motB3* *motA3*	Chemotaxis protein CheW Chemotaxis protein CheA Chemotaxis protein MotB Flagellar motor protein MotA	EAMY_2650 EAMY_2651 EAMY_2652 EAMY_2653	1.64E-04	2.313
*cheB1* *cheR1*	Chemotaxis response regulator protein-glutamate methylesterase Chemotaxis protein-glutamate O-methyltransferase	EAMY_2088 EAMY_2089	3.39E-05	3.227
*cheR3* *cheB3*	Chemotaxis response regulator protein-glutamate methylesterase Chemotaxis protein-glutamate O-methyltransferase	EAMY_2696 EAMY_2697	2.61E-07	8.574
*cheY1*	Response regulator	EAMY_2087	1.12E-03	3.681
*flgA1* *flgB1* *flgC1* *flgG1* *flgH1* *flgI1* *flgJ1*	Flagellar basal body P-ring biosynthesis protein FlgA Flagellar basal body rod protein FlgB Flagellar basal body rod protein FlgC Flagellar basal body rod protein FlgG Flagellar basal body L-ring protein Flagellar basal body P-ring protein Flagellar rod assembly protein/muramidase FlgJ	EAMY_1452 EAMY_1453 EAMY_1454 EAMY_1458 EAMY_1459 EAMY_1460 EAMY_1461	1.32E-03 1.88E-05 4.89E-04 6.72E-04 3.03E-05 3.14E-03	3.580 6.364 4.028 3.891 5.502 2.497
*flgM* *flhB3* *flhA3* *flhE3*	Anti-sigma-28 factor FlgM Flagellar biosynthesis protein FlhB Flagellar biosynthesis protein FlhA Flagellar biosynthesis protein FlhE	EAMY_1451 EAMY_2700 EAMY_2701 EAMY_2702	3.56E-04 3.00E-07	−4.084 3.655
*flhC1* *flhD1*	Transcriptional activator FlhC Flagellar transcriptional activator FlhD	EAMY_2099 EAMY_2100	2.81E-04	3.272
*flhD3*	Transcriptional activator FlhD	EAMY_2655	4.84E-06	−7.945
*fliD3*	Flagellar hook protein FliD	EAMY_2674	1.58E-03	4.408
*fliE1*	Flagellar hook-basal body complex protein FliE	EAMY_1512	7.67E-11	20.252
*fliI1* *fliH1* *fliG1* *fliF1*	Flagellum-specific ATP synthase FliI Flagellar assembly protein FliH Flagellar motor switch protein FliG Flagellar M-ring protein FliF	EAMY_1508 EAMY_1509 EAMY_1510 EAMY_1511	4.54E-04	5.205
*fliL1*	Flagellar basal body-associated protein FliL	EAMY_1505	2.80E-03	3.630
*fliP1* *fliO1*	Flagellar biosynthetic protein FliP Flagellar biosynthetic protein FliO	EAMY_1501 EAMY_1502	1.03E-05	4.532
*fliR3* *fliQ3*	Flagellar biosynthetic protein FliR Flagellar export apparatus protein FliQ	EAMY_2682 EAMY_2683	6.16E-12	−11.876
*fliS1*	Flagellar protein FliS	EAMY_2143	6.07E-04	3.945
*fliT1*	Flagellar biosynthesis protein FliT	EAMY_2144	7.21E-04	3.918
*fliZ*	Flagellar regulatory protein FliZ	EAMY_2138	2.41E-12	32.447
Iron acquisition				
*dfoJ*	Cytochrome c biogenesis protein CcmH	EAMY_3238	1.60E-05	-6.869
Type III secretion systems				
*hopPtoC*	Cysteine protease	EAMY_0744	8.19E-06	-6.589
*hrpF* *hrpG* *hrcC*	HPr kinase Hypothetical protein EscC/YscC/HrcC family type III secretion system outer membrane ring protein	EAMY_0547 EAMY_0548 EAMY_0549	2.05E-04	−2.888
*hrcJ* *hrpD* *hrpE*	EscJ/YscJ/HrcJ family type III secretion inner membrane ring protein Hypothetical protein Type III secretion system protein	EAMY_0544 EAMY_0545 EAMY_0546	4.80E-05	−2.809
*hrpA1*	Hrp pili protein HrpA	EAMY_0542	4.74E-03	−4.500
*hrpK*	Pathogenicity locus protein hrpK	EAMY_0519	6.02E-05	6.821
*hsvA*	Hypothetical protein	EAMY_0520	1.99E-03	−3.706
*invA3* *invB*	Type III secretion system protein InvA Hypothetical protein	EAMY_1580 EAMY_1581	8.99E-06	4.028
*spaT1* *sipB1*	CesD/SycD/LcrH family type III secretion system chaperone Type III secretion system protein	EAMY_0789 EAMY_0790	4.20E-07	−4.408
*spaI1* *spaM1* *spaN1* *spaO1* *spaP1* *spaQ1*	ATP synthase SpaL Hypothetical protein Hypothetical protein Type III secretion system protein EscR/YscR/HrcR family type III secretion system export apparatus protein Type III secretion system protein SpaQ	EAMY_0781 EAMY_0782 EAMY_0782 EAMY_0784 EAMY_0785 EAMY_0786	8.13E-06	−3.074
*spaK*	Hypothetical protein	EAMY_0780	4.73E-04	7.835
*spaS3*	EscU/YscU/HrcU family type III secretion system export apparatus switch protein	EAMY_1589	6.35E-04	4.287

Genes were considered as significantly differentially expressed, when their fold change was ≥1.5 or ≤ −1.5, respectively, and their *p* value <0.001

### The effect of T6SS deletion on plant pathogenicity

The transcriptomic data indicated that there is an effect of deletion of the T6SSs on pathogenicity-related gene expression. To get a first indication if T6SS is involved in pathogenicity of *E*. *amylovora* CFBP 1430 *in planta*, an immature pear assay was performed. The wildtype strain CFBP 1430, the single mutants and the double mutant were assessed for altered virulence on immature pear fruits. Additionally, a Δ*hrpL* mutant [44] was used as a positive control for altered pathogenicity. The alternative sigma factor HrpL controls the expression of different *hrp* genes and deletion thereof renders the mutant avirulent [44]. Strain CFBP 1430 and the T6SS mutants were applied at two different concentrations and were assessed at 8 DPI. The application of 10^7^ and 10^5^ cfu ml^−1^ led to similar tissue maceration and ooze production of all T6SS mutants compared to *E*. *amylovora* CFBP 1430 (Additional file 1: Figure S4). Symptoms could not be observed for any of the strains when 10^3^ cfu ml^−1^ was applied. The Δ*hrpL* mutant induced no symptoms at any concentration. The inoculation with *E*. *amylovora* CFBP 1430 and its T6SS mutant derivatives showed no difference in disease and symptom development.

**Fig. 2:**
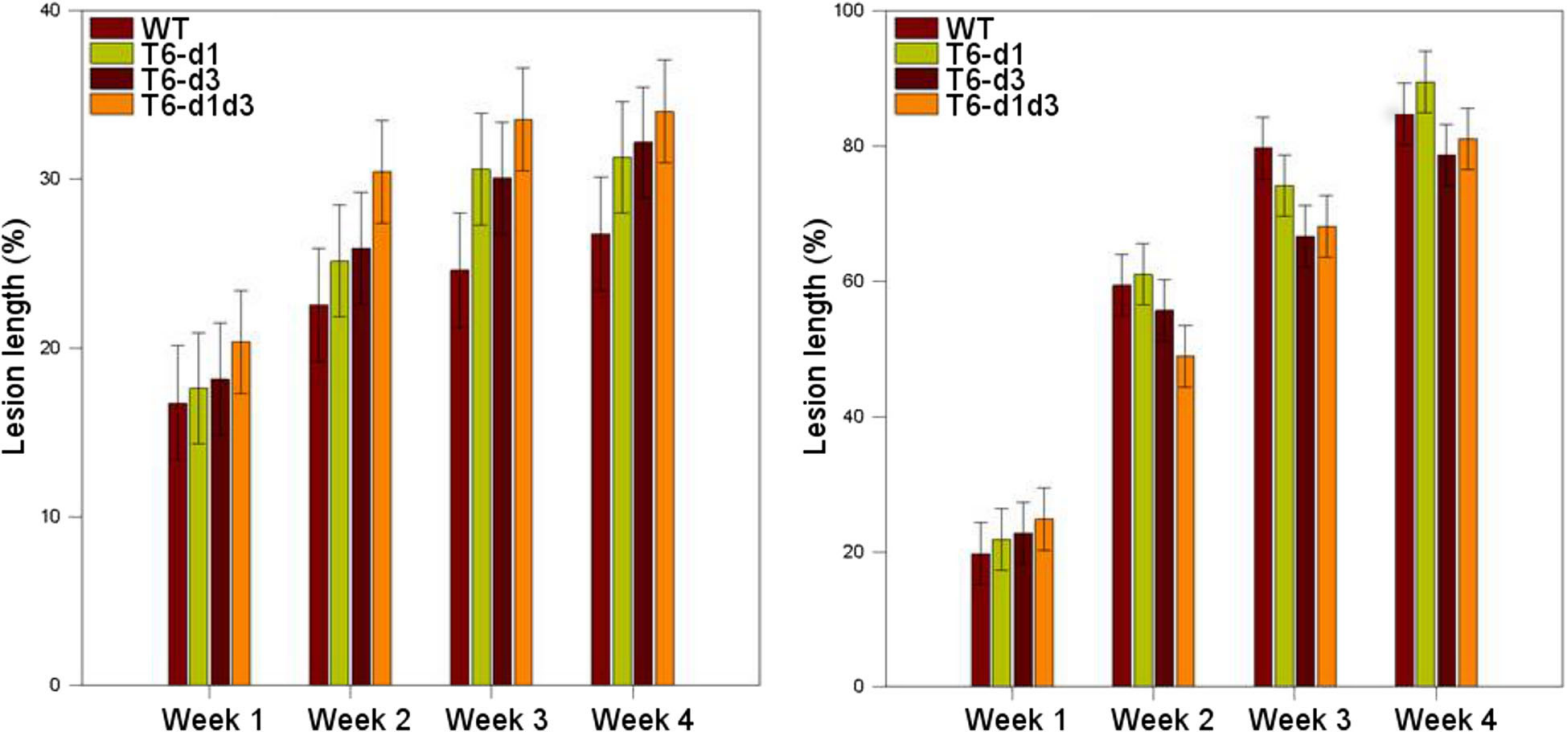
The effect of T6SS on virulence on apple (*left*) and pear (*right*) plants. Shoots were inoculated with a bacterial suspensions containing approximately 10^8^ cfu ml^−1^ of a single strain derivative. Lesion lengths were measured at weekly intervals over a 4-week period post inoculation. Values of percent lesion length represent the means of 25 plants with standard deviation. A two-way ANOVA with mean comparisons using the Fisher’s LSD test at a significance level of 5% was used to test for statistical significant differences. The T6SS double mutant showed higher lesion length in apple during the time course, whereas no differences to the WT could be detected in pear

To test for altered pathogenicity in apple and pear shoots, plants were inoculated either with CFBP 1430 or one of the three T6SS mutants. Disease development as well as disease progression in pear shoots showed no significant difference between CFBP 1430 and the T6SS mutants. In apple shoots, a significant treatment effect was observed, however, a significant effect of time point and treatment was not detected. Nevertheless, the double mutant T6-d1d3 exhibited a significant increase in necrotic lesion length compared to the CFBP 1430 (Fig. 2). Both single mutants, T6-d1 and T6-d3, showed no significant alteration in lesion formation. Percentage lesion length caused by the T6SS double mutant was highest throughout the experiment. The increase of necrotic lesion development depended on the mutation of both T6SS-1 and T6SS-3.

**Fig. 3:**
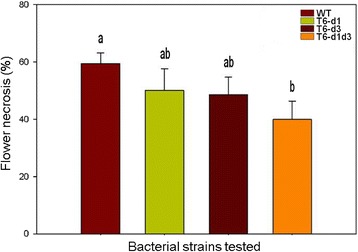
Role of T6SS in disease development on apple flowers. Flowers were inoculated with a bacterial suspensions containing approximately 10^7^ cfu ml^−1^ of a single strain derivative. Bars with standard deviation represent the means of two independent experiments. A one way ANOVA was applied with the Fisher’s LSD test at a significance level of 5% to test for statistical significant differences between treatments

Flowers are the primary infection court of *E*. *amylovora* in nature. The pathogen is able to invade host plants through the nectaries [45], thus no wounding of plant tissue as with artificial inoculation occurs. Freshly opened apple flowers of blooming trees were inoculated with bacterial suspensions containing either CFBP 1430 or one of the T6SS mutants. To check for altered growth patterns, the population sizes were determined at 1 DPI and 2 DPI. No significant differences were detected between the CFBP 1430 and the T6SS mutants at 1 DPI (Additional file 1: Figure S5). At 2 DPI the WT showed a statistical significant increase in cfu. Nevertheless, this difference was considered as biological irrelevant as the cfu of the WT and the mutants were in the same range. Scored at 10–12 DPI, the flowers showed a significant reduction of around 20% necrotic lesion for the T6-d1d3 compared to the WT (Fig. 3). The T6-d1 and T6-d3 single mutants showed a slight, but significant reduction of about 5% necrotic tissue. In other bacteria like *Legionella pneumophila*, *Salmonella enterica* subsp*. enterica* serovar. Typhimurium and *P. atrosepticum*, mutations in T6SS genes affected intracellular growth and led to altered replication rates [28, 46, 47]. We excluded that the effects on virulence were caused by differential replication rates, since all the T6SS mutants had similar growth rates in vitro and grew to same cell densities *in planta* (Additional file 1: Figures. S2 and S5). The influence on virulence was moderate compared to main factors like the Hrp-type III secretion system and the exopolysaccharide amylovoran [48, 49].

### Comparison of the *in planta* transcriptomes of *E. amylovora* CFBP 1430 and the T6SS double mutant

The transcriptome data derived from flowers at 2 DPI of the susceptible *Malus* × *domestica* cv. ‘Golden Delicious’ inoculated with either the wildtype *E*. *amylovora* CFBP 1430 or double mutant T6-d1d3. Alignment of the reads to the *E*. *amylovora* CFBP 1430 genome [34], showed that only 10.65% (CFBP 1430 inoculated) and 20.77% (T6-d1d3 inoculated sample) were mapped to the genome, probably due to cDNA derived from the apple host plants. This was confirmed by mapping the reads to the apple genome sequence [50] revealing that approximately 86.92% (WT inoculated sample) and 77.28% (T6-d1d3 inoculated sample) originated from apple RNA.

The bacterial transcriptome included 3563 expressed genes. Transcripts of 141 genes were found in none of the conditions, most encoding hypothetical proteins and five with annotations (*flgB3*, *glcG*, *intS1*, *spaR3*, *yeeV*). Among the non-expressed genes a flagellar (*flgB3*), a fimbrial (EAMY_0247) and a type III secretion system (*spaR3*) gene were identified. These genes were expressed in vitro (only *intS1* differentially). The differential expressed genes were grouped according to their GO annotations (Additional file 1: Figure S3). The biological processes include the categories cellular process, metabolic process, pigmentation, response to stimulus, biological regulation, and localization and locomotion.

*In planta* the T6-d1d3 mutation led to the significant differential expression of 56 genes, of which 49 are annotated while seven hypothetical genes were included (Additional file 1: Table S3). The T6SS mutations influence the processes of phosphate transport (*pstA3*, *pstC3*, *pstS3*), sulfur metabolism (*cysC*, *cysI3*, *cysJ*, *cysN*, *tauA*, *tauB*, *tauC*, *tauD3*), chemotaxis (*cheA1*, *cheB1*, *cheR1*, *cheY1*, *cheZ1*), and cell motility (*motA1*, *motB1*) as respective genes were identified to be differentially expressed (Table 3). All chemotaxis, cell motility, as well as the transport and sulfur metabolism genes had lower transcript abundance in double mutant T6-d1d3 compared to wildtype strain CFBP 1430. Some of these genes were differentially expressed in the in vitro transcriptome as well (*cheB1*, *cheR1*, *cheY1*, *motA1*, and *motB1*).

**Table 3:** Summary table of selected significantly differentially expressed genes from in vivo experiment comparing the WT to the T6SS double mutant

Name	Description	Fold change	Locus tag	*p* value
Chemotaxis and motility				
*cheZ1*	Protein phosphatase CheZ	−1.58185	EAMY_2086	4.64E-04
*cheY1*	Response regulator	−1.83835	EAMY_2087	3.44E-06
*cheB1* *cheR1*	Chemotaxis response regulator protein-glutamate methylesterase Chemotaxis protein-glutamate O-methyltransferase	−1.54751	EAMY_2088 EAMY_2089	3.55E-06
*cheA1*	Chemotaxis protein CheA	−1.56953	EAMY_2095	4.19E-04
*motB1* *motA1*	Flagellar motor protein MotB Flagellar motor protein MotA	−1.43199	EAMY_2096 EAMY_2097	9.34E-05
*tsr3*	Methyl-accepting chemotaxis protein	−1.56906	EAMY_2093	5.40E-04
*tsr7*	Methyl-accepting chemotaxis protein	−1.66575	EAMY_3131	7.23E-05
*fliC1*	Flagellin	−2.30592	EAMY_2141	4.65E-13
Phosphate transport				
*pstA3* *pstC3*	Phosphate transporter permease subunit PtsA Phosphate transporter permease subunit PstC	−2.37813	EAMY_3692 EAMY_3693	6.29E-05
*pstS3*	Phosphate ABC transporter substrate-binding protein	−2.31073	EAMY_3694	2.01E-05
*pspA*	Phage shock protein PspA	−1.73698	EAMY_1877	3.13E-05
Sulfur metabolism				
*tauD3* *tauC* *tauB*	Taurine dioxygenase Taurine ABC transporter permease Taurine ABC transporter ATP-binding protein	−2.15530	EAMY_3404 EAMY_3405 EAMY_3406	4.59E-07
*tauA*	Taurine ABC transporter substrate-binding protein	−1.95397	EAMY_3407	3.30E-04
*cysJ* *cysI3*	Sulfite reductase subunit alpha Sulfite reductase subunit beta	−1.46844	EAMY_0747 EAMY_0748	4.80E-05
*cysN* *cysC*	Sulfate adenylyltransferase Adenylyl-sulfate kinase	−1.87812	EAMY_0754 EAMY_0755	2.92E-09

Genes were considered as significantly differentially expressed, when their fold change was ≥1.4 or ≤ −1.4, respectively, and their *p* value <0.001

### In vitro experiments

**Fig. 4:**
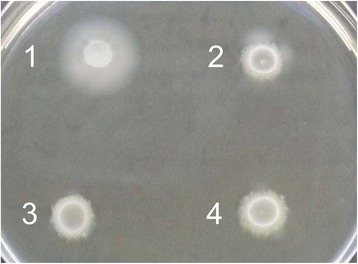
Influence of the T6SS mutations on cell motility. The wild-type and T6SS mutants were spotted on a motility agar and the diameters of the bacterial movement halos were assessed after 2 days post inoculation. (1) WT, (2) T6-d1, (3) T6-d3, (4) T6-d1d3

Both in vitro and *in planta* transcriptome data implied that chemotaxis and motility might be altered in the double mutant T6-d1d3. To test for a phenotypic effect, a motility assay was performed including the single T6SS mutants T6-d1 and T6-d3 and the strain CFBP1430. The T6-d1 mutant showed an asymmetric growth, while the T6-d3 and T6-d1d3 mutants showed no motility at 2 DPI, in contrast to wildtype CFBP 1430 (Fig. 4). The differences in motility did not arise from differential growth rates, as all strains grew at similar rates and to similar densities in the liquid media (Additional file 1: Figure S2).

To test for antibacterial properties of the T6SSs in *E*. *amylovora* CFBP 1430, a competition assay was performed challenging the WT and the T6SS mutants with *E. coli* DH5α on two different media. After co-inoculation with the WT and the single mutants on KB plates similar numbers of cfus were counted for *E. coli* DH5α. However, co-inculation with the double mutant resulted in slightly higher, statistically significant, numbers of *E. coli* DH5α cfus (Additional file 1: Figure S6). The competition assay on LB plates resulted in similar numbers of *E. coli* DH5α cfus.

## Discussion

In this study, a combined approach of RNA-seq, physiological tests and plant experiments was employed to analyze the impact of T6SS deletion on virulence of *E*. *amylovora* CFBP 1430. In contrast to the well-established virulence factors like the Hrp type III secretion and amylovoran [25, 44, 49, 51, 52], deletion of the T6SS did not impair the virulence of *E*. *amylovora* CFBP 1430 very strongly*.* Nevertheless, an alteration in virulence could be observed in apple flowers and shoots. This effect might be attributed to the impairment on motility that was displayed by the T6SS mutants, similarly to an *E*. *amylovora* motility mutant that showed reduced blossom infections [53]. On flowers, it is required to actively gain access to the vascular system through the nectartodes [54, 55]. Hence, the impaired T6SS mutants would produce less disease symptoms on flowers compared to the WT. When injected in the shoots, the T6SS mutants might be passively transported, as attachment structures (e.g., flagella, pili) would be less produced and therefore disease progress appeared faster. Alternatively, the identified differences on apple host plants were indicative that T6SS are involved in the control of disease progression and severity. Flagellar genes were also contained in the comparison of the *in planta* transcriptomes of the two strains, indicating that this trait is effectively affected in the double mutant. Motility of *E*. *amylovora* was demonstrated to be an important trait to invade non-injured apple leaves, whereas no significant effect could be detected on injured plants [56], and several changes in regulatory systems have an influence on motility [57]. Similarly, a difference in the expression of flagellar genes was also observed in T6SS mutants of *Citrobacter freundii* [58]*.* There, the deletion of the complete T6SS cluster led to a lower expression of all tested flagellar genes, whereas the deletion of *clpV*, *hcp*, and *vgr* showed both higher and lower expression levels dependent on the deleted gene [58]. As a direct impact of T6SSs genes on transcriptional changes is not expected, the absence of T6SS machinery might cause a change in the membrane properties or the accumulation of T6SS related proteins might lead to a deregulation and might affect transcription of the corresponding genes.

The comparison of the transcriptomic data obtained from the in vitro cultured wild-type strain CFBP 1430 and double mutant T6-d1d3 showed that virulence associated genes, e.g., Hrp T3SS associated, amylovoran biosynthesis, as well as a set of flagellar genes were affected by the deletion of the T6SS genes (Table 2). The differential expressed amylovoran biosynthesis genes *amsJ* and *amsK* might be involved in annealing the different galactose, glucuronic acid, and pyruvyl subunits while *amsL* in oligosaccharide transport and assembly [59]. The differential expression of these genes in the double mutant T6-d1d3 might lead to varying structure of the exopolysaccharide as annealing and assembly were altered. A difference in amylovoran production was observed for *E. amylovora* Ea1189 in a two-component signal transduction systems analysis, where deletion of the gene *spk1*, encoding the serine kinase corresponding to *ppkA* (EAMY_3004) in *E. amylovora* CFBP 1430 and thus part of T6SS-1 showed elevated level of the exopolysaccharide [57]. Additionally, single gene deletions in all three T6SSs had either no effect, increased or decreased exopolysacharide production [36]. The deletion of *tssB-1* and *tssC-1* increased, whereas *tssB-2* and *tssC-2* decreased the amylovoran production in *E*. *amylovora* NCPPB 1665 [36]*.*

The genes of the Hrp T3SS, beside the potential translocator encoding *hrpK*, were less abundant in the double mutant T6-d1d3 including *hsvA* and *hrpA*. The latter two genes are required for full virulence and systemic infection in apple plants [43, 52, 60]. The lower expression of these genes in the double mutant in vitro indicated that mutation of T6SS might lead to an altered virulence of the pathogen. The comparison with the in vivo transcriptomic data showed that only few genes were differentially expressed under both conditions and that the pathogenicity related were not part of them. Hence the differential expression of these genes was dependent on environmental factors.

The immature pear assays showed that no phenotypic difference was obtained between wildtype strain CFBP 1430 and all mutants, indicating that the T6SS is not directly involved in virulence on immature pear. A similar result was obtained for *E*. *amylovora* Ea1189 where deletion of *spk1* also did not show an effect on pathogenicity [57]. Single deletion mutants of *E*. *amylovora* NCPPB 1665 *tssB-1* and *tssC-1* showed as well no effect, whereas *tssB-2* and *tssC-2* decreased virulence of the pathogen on immature pears [36]. All three mutants generated in this study showed the same phenotype as the mutants in *tssB-1* and *tssC-1.* The difference to our results in the immature pear assay might arise from deletion of the consecutive genes *tssB*/*tssC* leading to a distinct phenotype. Additionally, different varieties of pear (‘Conférence’ vs. ‘Cuiguan’) as well as *E*. *amylovora* strains (CFPB 1430 vs. NCPPB 1665) were used to test for variation in virulence. The level of disease severity is dependent on both the *E*. *amylovora* strain and the genotype of host plant used to perform such experiments [61–63]. Furthermore, the differing results in the competition assay might arise as well from the *E*. *amylovora* strains used to test for antibacterial properties. As different *E*. *amylovora* strains are defined as ‘strong’ or ‘weak’ in respect to disease severity [61, 62], strains might exist that exhibit a similar pattern for interbacterial competition. Thus, *E*. *amylovora* strain NCPPB 1665 might be a better competitor than CFPB 1430 as mutational analysis of several genes in the T6SSs showed a stronger effect on survival of *E. coli* DH5α [36].

As *E*. *amylovora* is able to elicit disease symptoms in different plant organs beside fruits, we analyzed potential effects of T6SS mutations in shoots and flowers. The mutations of T6SSs genes in *E*. *amylovora* CFBP 1430 influenced the virulence in apple shoots, whereas the mutants showed no alteration on pear shoots or on immature pears. These results indicate that the T6SSs may contribute to virulence on apple, whereas on pear, the T6SSs were not strictly required to elicit disease symptoms. Additionally, these effects were dependent on the plant organ affected and thus T6SSs might be involved in adaptation, establishment and persistence of the pathogen to different host environments. This was further supported by the transcriptomic data obtained from in vitro and in vivo experiments that showed transcriptional changes were dependent on the environment. In the plant pathogenic bacterium *A. tumefaciens*, similar effects were identified from the deletion of *hcp*, that led to a reduced tumorgenesis in plant assays, whereas in *P*. *atrosepticum*, deletion mutants showed increased or slightly reduced virulence dependent on the deleted gene [7, 28].

As no visible effect of deletion of the T6SS on virulence in pears was obtained, the T6SSs might thus contribute as a host-specificity factor to the adaptation and control of disease progression. Further, the T6SSs in *E. amylovora* CFBP 1430 influenced only virulence on apple host plants, whereas virulence on pear was unaffected. Thus, the T6SSs might represent a host-specificity factor required to either promote or limit disease elicitation on apple shoots and flowers. It was observed that there is genetic variation of the T6SS gene clusters detected for *E. amylovora* strains infecting Spiraeoideae compared to *E. amylovora* strains infecting *Rubus* [64], which supports the hypothesis that the T6SSs could represent a potential role in host-specificity. Additionally, the closely related pear pathogens *Erwinia pyrifoliae* DSM 12163^T^ and *Erwinia pyriflorinigrans* CFBP 5888^T^ and the epiphyte *Erwinia tasmaniensis* Et1/99 possess only the two homologous T6SS gene clusters 1 and 2, while the third cluster is absent from their genome sequences [35, 65, 66].

The transcriptomic data obtained from flower inoculation experiments additionally showed that some metabolic processes were affected, namely phosphate transport and sulfur metabolism. Under the given conditions the genes of these pathways had lower transcript abundance in the double mutant T6-d1d3 than in the wildtype indicating that the T6SSs have an influence on metabolic functions on apple flowers. Sulfur and phosphate are two essential nutrients for growth and cell functions [67], but the lower expression of these genes did not significantly alter flower colonization.

## Additional file

Additional file 1: This PDF file contains all supplementary tables and supplementary figures listed below. **Table S1**. Primers used in this study. **Figure S1**. Qualitative RT-PCR of selected T6SS genes. **Figure S2**. Growth curves with standard deviation measured for *E*. *amylovora* CFBP 1430 WT, T6-d1, T6-d3, and T6-d1d3 for 48 h in different media. **Table S2**. Significantly differentially expressed genes from the in vitro experiment. **Figure S3.** Classes of *Erwinia amylovora* CFBP 1430 genes (GO terms) that were affected by the mutation of T6SS (comparing T6-d1d3 mutant to WT) representing the fire blight T6SS transcriptome. **Figure S4**. Immature pears inoculated with *E*. *amylovora* CFBP 1430 or one of the T6SS mutants. **Figure S5**. *E*. *amylovora* CFBP 1430 and T6SS mutant populations on apple flowers at 1 DPI and 2 DPI. **Table S3**. Significantly differentially expressed genes from the *in planta* experiment. **Figure S6.** Role of *E*. *amylovora* CFBP 1430 T6SSs in bacterial competition. (PDF 633 kb)
