# Supplementary material for: Role of the type VI secretion systems during disease interactions of Erwinia amylovora with its plant host
Source: BMC Genomics. 2017 Aug 17;18:628. doi: 10.1186/s12864-017-4010-1 (PMC5561584; doi:10.1186/s12864-017-4010-1)

Supplemental material belonging to:

Role of the type VI secretion systems during disease interactions of *Erwinia amylovora* with its plant host

Tim Kamber, Joël F. Pothier, Cosima Pelludat, Fabio Rezzonico, Brion Duffy and Theo H.M. Smits

Table S1. Primers used in this study.

Figure S1. Qualitative RT-PCR of selected T6SS genes.

Figure S2. Growth curves with standard deviation measured for *E. amylovora* CFBP 1430 WT, T6-d1, T6-d3, and T6-d1d3 for 48h in different media.

Table S2. Significantly differentially expressed genes from the *in vitro* experiment.

Figure S3. Classes of *Erwinia amylovora* CFBP 1430 genes (GO terms) that were affected by the mutation of T6SS (comparing T6-d1d3 mutant to WT) representing the fire blight T6SS transcriptome.

Figure S4. Immature pears inoculated with *E. amylovora* CFBP 1430 or one of the T6SS mutants.

Figure S5. *E. amylovora* CFBP 1430 and T6SS mutant populations on apple flowers at 1 DPI and 2 DPI.

Table S3. Significantly differentially expressed genes from the *in planta* experiment.

Figure S6. Role of *E. amylovora* CFBP 1430 T6SSs in bacterial competition.

Table S1. Primers used for mutant generation and RT-PCR

Description	Primer sequence	Length (bp)	Annealing temperature
T6-d1-F	ATGGAGAACACCATGGCAGTCAGTAAATCCAGTGGGCAGAAATTCATTGCgtgtaggctggagctgcttc	2002	55°C
T6-d1-R	TCACGCGTCGTTCGATTTCAGCGACGGCAGTTTAGAAACCAGACGCAGTGcatatgaatatcctcctta		
T6-d3-F	ATGTCTAACTCCTGGCAGTCCGAGATCCCCAAGGCTCGCGTTAACATTCAgtgtaggctggagctgcttc	2030	55°C
T6-d3-R	TTATGCCTTGGCTTTTGGCATCTGCGACACCAGCGACAGATTGACGTCCAcatatgaatatcctcctta		
hcp1-F	ACACCGGTTGGATCGACGTGTCT	363	64°C
hcp1-R	CGGAGGTGCTCTGCTCCCAGTAC		
hcp2-F	TCTCTCGCAGGATCATAGCCTGT	181	57°C
hcp2-R	GATTCTGTACCACTTGAACTCGG		
recA-F	CCATCATGCGCCTGGGTGAAGAC	213	64°C
recA-R	CGGCATCGATAAACGCACAGGTC		
duf879-1-F	GAGATGATTGCGCCGAACTACCT	179	64°C
duf879-1-R	GTACCGGTTGCAGCATCACATCG		
vgrG4-F	AGCTGACCAGTCGCACTACACAA	379	57°C
vgrG4-R	TTCCGCACCGTCGAACAACCCAG		
duf879-2-F	GACCCGTACGTCGAACGCCTGTT	204	64°C
duf879-2-R	CTCCGACAGACACTCAGACTGGC		
ffh-F	CGATAGACTATCGCAAACCCTG	177	57°C
ffh-R	TTCACTTCCTGACCAACGGC		

Figure S1. Qualitative RT-PCR of selected T6SS genes and housekeeping genes. (1) *recA* (2) *tssF-1* T6SS cluster 1 (3) *hcp-1* T6SS cluster 1 (4) *tssF-2* T6SS cluster 3 (5) *hcp-2* duf879 T6SS cluster 3 (6) *ffh* (7) *vgrG-4* T6SS cluster 3. RNA was extracted from *E. amylovora* CFBP 1430 grown in different media. KB=King's B medium, Hrp Suc/Hrp sorb=Hrp minimal medium either supplemented with sucrose or sorbitol.

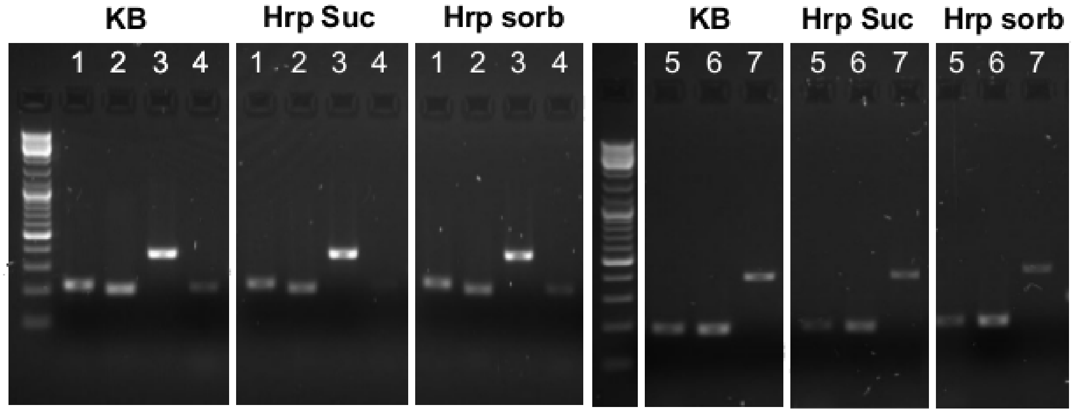

Figure S2. Growth curves with standard deviation measured for *E. amylovora* CFBP 1430 WT, T6-d1, T6-d3, and T6-d1d3 for 48h in different media. A) King's B medium, B) M9 minimal medium supplemented with sucrose, C) synthetic nectar, D) Hrp minimal medium supplemented with sucrose. The growth rates of the WT and the T6SS mutants in the different media is summarized in the table.

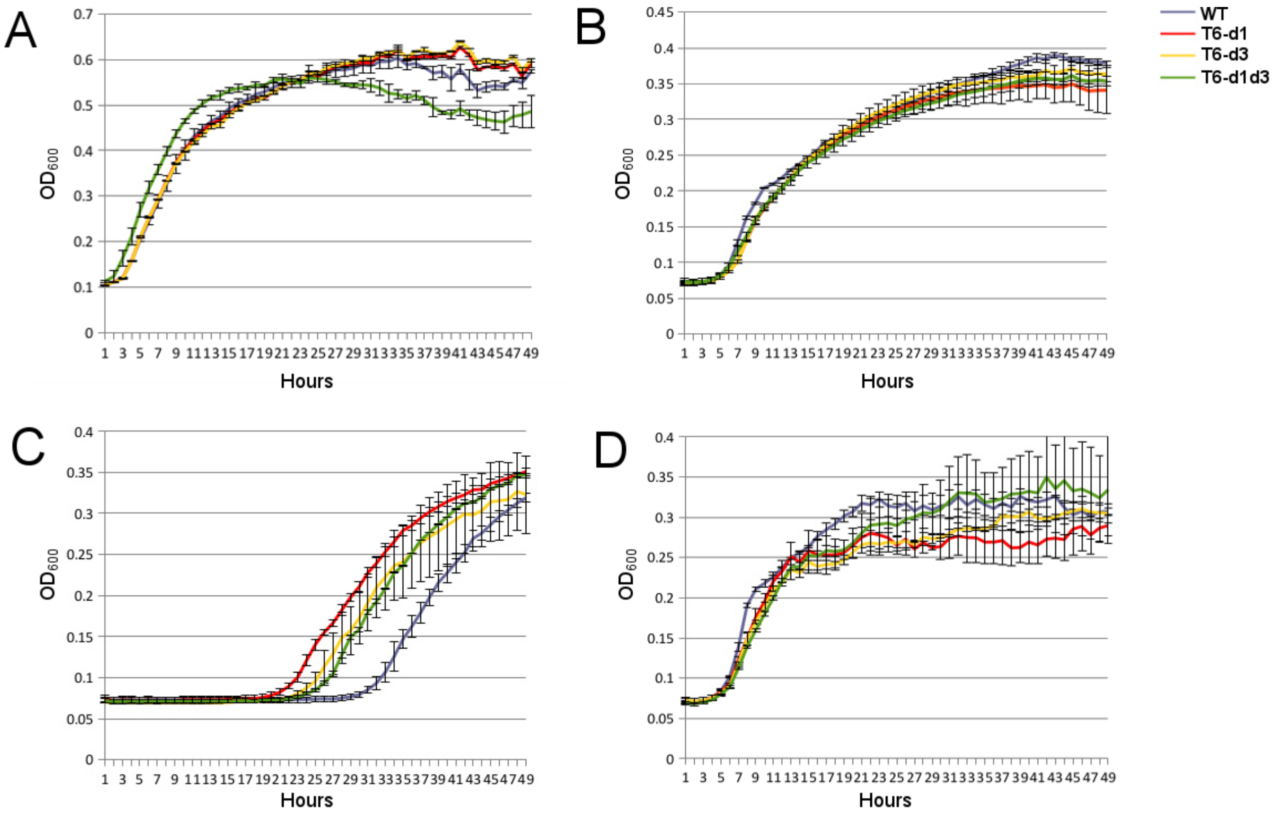

	KB	M9 sucrose	Synthetic nectar	HrpMM sucrose
**WT**	0.00454	0.00582	0.01345	0.01910
**T6-d1**	0.00407	0.00546	0.01305	0.01842
**T6-d3**	0.00429	0.00546	0.01776	0.01842
**T6-d1d3**	0.00473	0.00522	0.01374	0.02016

Table S2. Significantly differentially expressed genes from the *in vitro* experiment

***E. amylovora* locus tag or name**	**locus tag**	**FPKM (WT)**	**FPKM (T6-d1d3)**	**Fold change**	**p value**
EAMY_1758	EAMY_1758	714717	426.136	-1677.15	0
EAMY_2041	EAMY_2041	10845.5	64.9974	-166.86	1.90844E-05
EAMY_1642	EAMY_1642	3329.25	38.2334	-87.08	7.93101E-09
relB	EAMY_2780	830.429	13.7505	-60.39	8.51477E-05
EAMY_3720	EAMY_3720	31026.8	530.957	-58.44	0
EAMY_2810	EAMY_2810	2057.9	39.1503	-52.56	0.000149655
EAMY_0058	EAMY_0058	18654.3	379.083	-49.21	6.19949E-12
EAMY_1490	EAMY_1490	36312.5	811.717	-44.74	2.0089E-11
EAMY_3336	EAMY_3336	32379.5	774.165	-41.83	7.55396E-13
EAMY_1595	EAMY_1595	3916.36	94.008	-41.66	0
intR	EAMY_1837	1667.98	47.4485	-35.15	1.22347E-13
EAMY_0296	EAMY_0296	31515.6	928.998	-33.92	4.9738E-13
intS1	EAMY_2565	16068.7	502.37	-31.99	1.82077E-14
rpmE2,rpmJ2	EAMY_1006,EAMY_1007	42272	1399.92	-30.20	0
EAMY_0766	EAMY_0766	5446.88	202.929	-26.84	0.00172809
EAMY_3568	EAMY_3568	6320.11	245.7	-25.72	0.00199696
EAMY_2017	EAMY_2017	18640.4	757.601	-24.60	1.01452E-12
EAMY_0604	EAMY_0604	3336.09	142.045	-23.49	0.0026677
EAMY_3614	EAMY_3614	3463.26	154.833	-22.37	0.00311368
kdpE	EAMY_1154	1290.48	59.2422	-21.78	1.81308E-11
EAMY_2539	EAMY_2539	7099.71	360.619	-19.69	4.60462E-06
ygdL	EAMY_0717	944.729	51.7179	-18.27	1.22387E-10
EAMY_1879	EAMY_1879	1.69874	94242.5	-18.03	3.41534E-07

		e+06			
EAMY_2553	EAMY_2553	1105.51	63.1489	-17.51	8.21986E-10
fldX	EAMY_1952	855.742	50.8223	-16.84	3.81708E-10
EAMY_3093	EAMY_3093	637.477	38.0975	-16.73	3.84033E-11
oppA5,oppB3,oppC3,oppD3	EAMY_2169,EAMY_2170,EAMY_2171,EAMY_2172	3669.68	264.037	-13.90	0
EAMY_3644	EAMY_3644	473.219	35.1333	-13.47	7.14049E-10
ydhB	EAMY_1688	1455.22	118.369	-12.29	0.000000085
mgsA	EAMY_1392	2943.36	240.164	-12.26	3.3482E-08
fliQ3,fliR3	EAMY_2682,EAMY_2683	1491.5	125.963	-11.84	6.15663E-12
pyrF,yciH	EAMY_1899,EAMY_1900	3777.62	323.227	-11.69	2.26316E-10
EAMY_0562	EAMY_0562	419.555	36.7274	-11.42	2.13641E-08
EAMY_0044	EAMY_0044	1711.63	151.009	-11.33	9.7726E-06
EAMY_1094,EAMY_1095	EAMY_1094,EAMY_1095	1214.69	107.252	-11.33	0.00265807
EAMY_3639	EAMY_3639	52112.9	4814.4	-10.82	6.24576E-08
yqeI	EAMY_3646	1081.1	103.571	-10.44	1.86467E-08
tas	EAMY_0693	1397.44	133.959	-10.43	6.72443E-07
elaA	EAMY_2357	655.495	64.2989	-10.19	2.38143E-08
yebY	EAMY_2036	4338.36	429.965	-10.09	1.39134E-07
EAMY_3056	EAMY_3056	21198	2179.73	-9.73	5.57701E-08
EAMY_1513	EAMY_1513	4671.38	491.4	-9.51	0.00395645
EAMY_0745	EAMY_0745	4329.07	464.499	-9.32	0.000720842
ybaV	EAMY_0988	626.077	67.5626	-9.27	3.53448E-07
orfB1,ybjR1	EAMY_1317,EAMY_1318	2931.97	318.348	-9.21	1.07237E-08
yfaE	EAMY_2349	1004.94	110.028	-9.13	5.28302E-07
EAMY_3029	EAMY_3029	9870.28	1083.83	-9.11	4.27979E-06
EAMY_3204	EAMY_3204	298.68	33.7344	-8.85	1.23948E-07

EAMY_2759	EAMY_2759	62750.2	7102.27	-8.84	1.5337E-07
EAMY_0490	EAMY_0490	964.928	111.327	-8.67	0.000250446
EAMY_2213	EAMY_2213	5673.47	676.388	-8.39	0.000317424
EAMY_3021	EAMY_3021	126.836	15.3776	-8.25	4.67179E-06
EAMY_3207	EAMY_3207	368.785	45.8779	-8.04	0.000001452
EAMY_2384	EAMY_2384	80185	9976.73	-8.04	4.69198E-07
flhD3	EAMY_2655	372.767	47.005	-7.93	4.84045E-06
ygfP	EAMY_1748	733.134	93.8955	-7.81	5.50755E-06
panF,yhdT	EAMY_0274,EAMY_0275	1745.81	224.948	-7.76	1.84897E-08
nlp3	EAMY_0410	485.289	64.1018	-7.57	5.05674E-06
EAMY_1076	EAMY_1076	3566.92	490.67	-7.27	0.000018735
cutC	EAMY_2078	988.792	139.352	-7.10	4.76947E-06
EAMY_2592	EAMY_2592	910.865	128.735	-7.08	3.10711E-06
yghA	EAMY_0472	454.953	65.311	-6.97	2.21024E-06
dfoJ	EAMY_3238	555.053	80.8039	-6.87	1.60241E-05
mrdA	EAMY_1118	471.342	69.8617	-6.75	2.30377E-05
HopPtoC	EAMY_0744	827.496	125.263	-6.61	8.19247E-06
EAMY_0795,EAMY_0796	EAMY_0795,EAMY_0796	3401.55	533.6	-6.37	0.000000186
EAMY_0859	EAMY_0859	2107.87	332.601	-6.34	0.00558966
ung	EAMY_2638	1466.66	234.276	-6.26	3.39405E-05
EAMY_2269	EAMY_2269	1133.44	181.194	-6.26	6.08355E-06
lipB	EAMY_1113	392.422	64.0207	-6.13	7.70971E-06
artP	EAMY_1315	288.881	47.7431	-6.05	1.01459E-05
fadA	EAMY_0222	554.332	92.0117	-6.02	2.41133E-05
alsD	EAMY_0013	429.813	72.198	-5.95	1.00642E-05
iolD	EAMY_3508	79.0116	13.3859	-5.90	1.39004E-05

EAMY_3423	EAMY_3423	160.619	27.6187	-5.82	0.00132212
EAMY_2076	EAMY_2076	353.712	63.2541	-5.59	1.92699E-05
EAMY_3186	EAMY_3186	39909.9	7137.05	-5.59	0.000028163
nrdH	EAMY_2709	1392.46	249.013	-5.59	4.47202E-05
EAMY_3260	EAMY_3260	955.226	173.201	-5.52	0.000171305
EAMY_2449,EAMY_2450, *rcsC3*	EAMY_2449,EAMY_2450,EAMY_2451	719.516	131.645	-5.47	5.14992E-10
EAMY_0485	EAMY_0485	821.603	155.042	-5.30	0.000276948
yqjA,yqjB	EAMY_3101,EAMY_3102	1014.85	192.461	-5.27	3.07207E-06
EAMY_1776	EAMY_1776	8305.53	1577.28	-5.27	7.71434E-05
yejH	EAMY_2319	217.901	42.0478	-5.18	4.65924E-05
yohN	EAMY_0801	4303.13	852.34	-5.05	0.000588955
prgH1	EAMY_0775	1001.2	199.207	-5.03	0.000647239
hmsT	EAMY_3035	332.216	66.3316	-5.01	5.76622E-05
yibK	EAMY_0103	636.378	131.495	-4.84	8.67387E-05
ygdR3	EAMY_1567	2569.37	533.097	-4.82	0.000106605
goaG	EAMY_2391	187.318	39.5884	-4.73	9.79802E-05
uvrB	EAMY_1211	564.117	119.539	-4.72	0.00101458
yfhD	EAMY_2611	454.039	96.4648	-4.71	0.000299874
sapA,sapB,sapC,sapD,sapF	EAMY_1880,EAMY_1881,EAMY_1882,EAMY_1883,EAMY_1884	1734.9	369.196	-4.70	1.47923E-06
EAMY_1519	EAMY_1519	437.023	94.0338	-4.65	0.000491703
EAMY_1929	EAMY_1929	33816	7305.46	-4.63	0.00017874
cysZ	EAMY_2483	599.234	131.358	-4.56	0.000155572
hrpA1	EAMY_0542	49210.6	10959.1	-4.49	0.00473651
EAMY_2969	EAMY_2969	531.254	119.806	-4.43	0.00108135
sipB1,spaT1	EAMY_0789,EAMY_0790	655.196	148.71	-4.41	4.1952E-07

EAMY_3589	EAMY_3589	9728.03	2230.33	-4.36	0.00188796
EAMY_3224,EAMY_3225, EAMY_3226	EAMY_3224,EAMY_3225,EAMY_3226	738.548	172.282	-4.29	1.85156E-05
ybiS	EAMY_1226	1038.33	246.478	-4.21	0.00208862
norM	EAMY_1684	512.509	123.526	-4.15	0.00101518
plc	EAMY_1766	726.721	177.569	-4.09	0.00115726
flgM	EAMY_1451	2476.22	606.69	-4.08	0.000356388
EAMY_0010	EAMY_0010	262.177	64.5484	-4.06	0.00554573
EAMY_3271	EAMY_3271	289.786	72.3988	-4.00	0.000688928
EAMY_0751	EAMY_0751	9690.24	2453.39	-3.95	0.000486343
ycaD	EAMY_1339	802.432	206.066	-3.89	0.00265391
EAMY_3022	EAMY_3022	204.874	52.9032	-3.87	0.000625594
prtF	EAMY_3577	193.807	50.3289	-3.85	0.000587648
EAMY_2997	EAMY_2997	2020.94	525.678	-3.84	0.00274733
tolQ	EAMY_1173	874.308	227.618	-3.84	0.00100183
ymcA,ymcB	EAMY_3333,EAMY_3334	742.709	193.504	-3.84	7.13073E-06
yebA	EAMY_2059	397.921	105.794	-3.76	0.00134354
yeaZ	EAMY_2006	346.729	93.3671	-3.71	0.000843571
yjeR	EAMY_3167	515.804	139.04	-3.71	0.000830988
hsvA	EAMY_0520	583.461	157.372	-3.71	0.00199425
yrbE	EAMY_0324	546.583	147.728	-3.70	0.000972159
ydcO	EAMY_1527	321.485	87.8334	-3.66	0.00104872
yhfC	EAMY_3419	312.422	85.4484	-3.66	0.00104172
yddG	EAMY_0506	334.55	91.9707	-3.64	0.000953914
mod	EAMY_0851	429.737	118.545	-3.63	0.00254275
ychH	EAMY_1602	3218.3	891.855	-3.61	0.00113782

EAMY_2092	EAMY_2092	639.928	179.5	-3.57	0.00406282
EAMY_3227	EAMY_3227	75.1959	21.1313	-3.56	0.00177085
yhdZ	EAMY_0263	195.878	55.3087	-3.54	0.00173734
sdaC	EAMY_1296	187.696	54.9471	-3.42	0.00162039
bolA	EAMY_0979	5590.79	1640.55	-3.41	0.00554345
ymcC	EAMY_3335	1433.77	420.868	-3.41	0.003357
EAMY_2386,*pta*	EAMY_2386,EAMY_2387	1059.2	313.501	-3.38	0.000352567
amiB,yjeE,yjeF	EAMY_3160,EAMY_3161,EAMY_3162	1339.96	396.647	-3.38	0.000117417
mreC,mreD	EAMY_0284,EAMY_0285	838.875	248.378	-3.38	0.000592649
EAMY_1991	EAMY_1991	20204.4	6021.89	-3.36	0.00437839
ybgR	EAMY_1183	358.109	108.212	-3.31	0.00215823
yneH	EAMY_1796	222.127	67.6892	-3.28	0.00243578
rpmG	EAMY_0078	15223.2	4684.82	-3.25	0.00253195
EAMY_3631,*dalD*	EAMY_3630,EAMY_3631	907.437	280.55	-3.23	0.000634029
EAMY_2957	EAMY_2957	1047.78	325.44	-3.22	0.00309035
EAMY_0405,*lcrS*	EAMY_0405,EAMY_0404	1359.98	423.3	-3.21	0.0034904
amsJ	EAMY_2244	409.017	128.497	-3.18	0.00484671
artJ	EAMY_1311	204.207	64.5155	-3.17	0.00410954
EAMY_2276	EAMY_2276	623.994	200.51	-3.11	0.00394261
spaI1,spaM1,spaN1,spaO1,spaP1,spaQ1	EAMY_0781,EAMY_0782,EAMY_0783,EAMY_0784,EAMY_0785,EAMY_0786	4399.76	1429.4	-3.08	8.13237E-06
aaeB	EAMY_0293	112.365	37.1706	-3.02	0.00426871
mesJ	EAMY_2740	259.502	86.2815	-3.01	0.00489644
yicG	EAMY_0053	251.272	83.6717	-3.00	0.00610846
yhiN	EAMY_3531	229.354	76.9036	-2.98	0.00471065
znuB,znuC	EAMY_2061,EAMY_2062	472.989	159.107	-2.97	0.000170043

EAMY_0645	EAMY_0645	603.935	204.284	-2.96	0.00516808
EAMY_0126	EAMY_0126	168.843	57.3203	-2.95	0.0059595
yieE	EAMY_3684	343.485	116.66	-2.94	0.00524874
livG,livH,livM	EAMY_3486,EAMY_3487,EAMY_3488	967.158	328.771	-2.94	0.000358889
ygcM	EAMY_0746	1007.32	343.314	-2.93	0.00564874
EAMY_1066	EAMY_1066	3531.79	1222.43	-2.89	0.00598703
hrcC,hrpF,hrpG	EAMY_0547,EAMY_0548,EAMY_0549	1955.79	677.938	-2.88	0.000204588
hrcJ,hrpD,hrpE	EAMY_0544,EAMY_0545,EAMY_0546	748.729	266.511	-2.81	4.80285E-05
EAMY_2193,*yviA*	EAMY_2193,EAMY_2194	1359.93	484.258	-2.81	0.00187419
pdxY1,pdxY3	EAMY_1708,EAMY_1709	302.325	114.329	-2.64	0.00289515
EAMY_2488,*cpxR3*	EAMY_2488,EAMY_2489	436.807	173.202	-2.52	0.00168251
gph,rpe,trpS	EAMY_3425,EAMY_3426,EAMY_3427	1690.76	705.848	-2.40	0.00443999
EAMY_3011,EAMY_3012	EAMY_3011,EAMY_3012	516.959	219.93	-2.35	0.00450181
EAMY_0112	EAMY_0112	1995.49	0	0.00	0.00233887
EAMY_0580	EAMY_0580	21158.6	0	0.00	0.000229489
EAMY_0670	EAMY_0670	1947.3	0	0.00	0.00132701
ygcB,ygcJ,ygcK,ygcL	EAMY_2817,EAMY_2818,EAMY_2819,EAMY_2820	284.306	512.618	1.80	0.00571181
dppD3,dppF	EAMY_3609,EAMY_3610	192.01	427.364	2.23	0.00490375
hisA,hisF,hisI	EAMY_2225,EAMY_2226,EAMY_2227	331.064	748.812	2.26	0.00118769
cheA3,cheW3,motA3,motB3	EAMY_2650,EAMY_2651,EAMY_2652,EAMY_2653	138.655	321.088	2.32	0.000164074
mdtA,mdtB,mdtC	EAMY_0683,EAMY_0684,EAMY_0685	174.56	408.677	2.34	0.00469217
rplP,rpmC,rpsQ	EAMY_3377,EAMY_3378,EAMY_3379	15273.3	36278.4	2.38	0.00402704
emrB1,hlyD	EAMY_1279,EAMY_1280	75.7195	189.12	2.50	0.0018841
flgI1,flgJ1	EAMY_1460,EAMY_1461	224.075	560.098	2.50	0.00314278
EAMY_3017,EAMY_3018	EAMY_3017,EAMY_3018	688.329	1732.85	2.52	0.004804
EAMY_3006,EAMY_3007	EAMY_3006,EAMY_3007	98.1019	265.373	2.71	0.00211331

EAMY_3008,EAMY_3009	EAMY_3008,EAMY_3009	168.508	463.793	2.75	0.000980378
proV,proW	EAMY_2713,EAMY_2714	108.247	301.353	2.78	0.000321945
ggt3	EAMY_0856	40.1946	119.022	2.96	0.00576223
yfhB	EAMY_2613	97.3479	292.111	3.00	0.00580983
holB,pabC,tmk,yceG	EAMY_1477,EAMY_1478,EAMY_1479,EAMY_1480	365.368	1103.05	3.02	0.000404829
yejB,yejE	EAMY_2312,EAMY_2313	299.582	908.878	3.03	0.00201863
yifA	EAMY_0155	159.139	488.748	3.07	0.00499348
sbcC,sbcD	EAMY_0936,EAMY_0937	90.1187	278.683	3.09	0.000229889
rpmB	EAMY_0077	655.377	2063.22	3.15	0.00447458
EAMY_3575	EAMY_3575	125.101	402.872	3.22	0.00328817
cheB1,cheR1	EAMY_2088,EAMY_2089	128.59	414.37	3.22	3.39484E-05
EAMY_3554,EAMY_3555	EAMY_3554,EAMY_3555	172.15	555.38	3.23	0.000518598
flhC1,flhD1	EAMY_2099,EAMY_2100	1006.43	3296.7	3.28	0.000281351
EAMY_2461	EAMY_2461	412.507	1364.72	3.31	0.00295338
ycgZ	EAMY_1569	292.839	970.995	3.32	0.0048046
EAMY_1249,EAMY_1250	EAMY_1249,EAMY_1250	58.2544	193.822	3.33	0.000479112
EAMY_3288	EAMY_3288	1370.12	4570.53	3.34	0.00421303
crcA	EAMY_1108	64.4618	215.385	3.34	0.00312893
gntK,gntU	EAMY_3472,EAMY_3473	155.707	521.685	3.35	0.00036569
trpI	EAMY_1020	40.7081	137.933	3.39	0.00279038
sulA	EAMY_1387	96.7612	329.193	3.40	0.00260466
EAMY_1063	EAMY_1063	199.402	683.464	3.43	0.00303988
yvrE	EAMY_2458	109.947	379.056	3.45	0.00181185
sufE	EAMY_1680	126.749	438.798	3.46	0.00231549
EAMY_0370	EAMY_0370	10476.3	36354.4	3.47	0.0021768
ychF	EAMY_1597	171.285	594.468	3.47	0.0039378

yeeO	EAMY_2212	44.4685	155.477	3.50	0.00156435
prgJ3,prgK3	EAMY_1574,EAMY_1575	101.242	354.304	3.50	0.00016528
EAMY_2707	EAMY_2707	168.984	602.097	3.56	0.00141019
flgA1	EAMY_1452	148.259	530.26	3.58	0.00132295
tap3	EAMY_2090	82.2753	294.265	3.58	0.00215072
ydbL	EAMY_1848	199.697	714.233	3.58	0.00183343
recQ	EAMY_0198	60.2966	218.035	3.62	0.00160925
fliL1	EAMY_1505	51.2073	185.437	3.62	0.00279698
EAMY_1759	EAMY_1759	23.5738	85.4849	3.63	0.00317007
hha	EAMY_1001	837.117	3040.8	3.63	0.00139378
yraL	EAMY_3113	266.161	967.385	3.63	0.00376473
yhhK	EAMY_3491	104.523	380.066	3.64	0.00185678
EAMY_0242	EAMY_0242	59.1797	215.311	3.64	0.00185841
flhA3,flhB3,flhE3	EAMY_2700,EAMY_2701,EAMY_2702	72.6701	265.357	3.65	3.00224E-07
EAMY_1270	EAMY_1270	53.9723	198.176	3.67	0.00113564
cheY1	EAMY_2087	224.953	828.429	3.68	0.00111699
rpmJ	EAMY_3365	8872.83	33176.9	3.74	0.000940137
polB	EAMY_2919	35.7972	134.912	3.77	0.000938957
EAMY_3341	EAMY_3341	1516.98	5777.25	3.81	0.00134781
speC	EAMY_0578	55.462	211.678	3.82	0.00125651
yciU	EAMY_1932	282.431	1082.29	3.83	0.000871457
waaG,waaQ3	EAMY_0085,EAMY_0086	95.6893	367.009	3.84	0.000020228
sdhA,sdhC,sdhD	EAMY_1164,EAMY_1165,EAMY_1166	1602.39	6156.54	3.84	0.000177419
EAMY_1539	EAMY_1539	416.784	1614.89	3.87	0.00297427
emrR	EAMY_2717	238.576	927.655	3.89	0.000752508
flgG1	EAMY_1458	75.7763	295.457	3.90	0.000672271

EAMY_1815	EAMY_1815	700.562	2740.52	3.91	0.00210414
fliT1	EAMY_2144	200.428	786.973	3.93	0.000720697
yicE	EAMY_0045	121.791	478.602	3.93	0.00169555
fliS1	EAMY_2143	218.472	862.777	3.95	0.000606669
EAMY_3729	EAMY_3729	322.831	1278.82	3.96	0.000568913
yfhH	EAMY_2615	33.9465	134.566	3.96	0.00087254
engA	EAMY_2575	129.212	516.918	4.00	0.00196559
yadB	EAMY_2789	62.3045	249.322	4.00	0.000535842
flgC1	EAMY_1454	268.658	1080.99	4.02	0.000489443
EAMY_3654	EAMY_3654	266.581	1074.07	4.03	0.00199647
yigQ	EAMY_0096	137.46	553.982	4.03	0.000770447
invA3,invB	EAMY_1580,EAMY_1581	96.0109	387.805	4.04	8.98522E-06
fadB	EAMY_0223	88.5392	358.369	4.05	0.00198146
pagO	EAMY_0991	46.4298	190.011	4.09	0.000469811
yhjJ1,yhjK1,yhjL	EAMY_0625,EAMY_0626,EAMY_0627	556.727	2283.11	4.10	0.000542064
amtB	EAMY_0996	104.47	428.87	4.11	0.000856243
EAMY_3696	EAMY_3696	60.3404	248.184	4.11	0.00269658
inh	EAMY_3580	190.888	787.034	4.12	0.000433184
acs	EAMY_3275	101.425	418.791	4.13	0.00179523
yeiE	EAMY_2300	74.0029	307.215	4.15	0.000374536
EAMY_2968	EAMY_2968	180.581	758.889	4.20	0.00111548
yhcA	EAMY_1862	28.6842	121.827	4.25	0.000698911
amsK,amsL	EAMY_2243,EAMY_2242	449.047	1909.77	4.25	0.00105348
spaS3	EAMY_1589	16.2355	69.6813	4.29	0.000634804
yqfA	EAMY_0646	198.399	870.093	4.39	0.000368138
fliD3	EAMY_2674	165.331	728.957	4.41	0.0015782

ptrB	EAMY_2046	85.7795	379.606	4.43	0.00096653
fpr	EAMY_0121	75.4697	335.037	4.44	0.000216462
EAMY_3088	EAMY_3088	359.847	1608.78	4.47	0.00366014
fliO1,fliP1	EAMY_1501,EAMY_1502	143.105	646.328	4.52	1.03429E-05
EAMY_3538	EAMY_3538	166.424	755.483	4.54	0.000172104
yeeS	EAMY_0398	24.0647	109.978	4.57	0.000750683
EAMY_1647	EAMY_1647	279.128	1289.5	4.62	0.00292309
motA1,motB1	EAMY_2096,EAMY_2097	347.878	1608.62	4.62	1.13577E-06
otsA1,otsB	EAMY_2102,EAMY_2103	560.375	2603.21	4.65	0.000634067
argD	EAMY_3415	170.706	793.07	4.65	0.000831973
EAMY_2953	EAMY_2953	1319.44	6166.99	4.67	0.00024895
htrA	EAMY_2765	300.436	1406.47	4.68	0.00236921
yqhC	EAMY_0464	109.935	521.249	4.74	0.00016655
EAMY_0373	EAMY_0373	42.5993	205.486	4.82	0.000114468
EAMY_1269	EAMY_1269	145.726	718.838	4.93	0.000629295
EAMY_0231	EAMY_0231	604.778	2994.98	4.95	0.000362688
ygbF,ygbT	EAMY_2813,EAMY_2814	176.842	875.99	4.95	1.71779E-07
citA	EAMY_0471	244.362	1219.31	4.99	0.00104245
ppx	EAMY_2548	241.707	1209.4	5.00	0.00125851
yejL	EAMY_2324	553.619	2777.59	5.02	7.96828E-05
gcvH	EAMY_0641	170.434	865.87	5.08	6.59298E-05
EAMY_1921	EAMY_1921	124962	644441	5.16	0.00354996
yidR	EAMY_3672	33.8343	175.893	5.20	6.97437E-05
fliF1,fliG1,fliH1,fliI1	EAMY_1508,EAMY_1509,EAMY_1510,EAMY_1511	1029.18	5354.01	5.20	0.000454094
ybeB	EAMY_1120	102.579	534.136	5.21	0.000136079
EAMY_2030	EAMY_2030	117.535	614.639	5.23	6.84146E-05

inlA	EAMY_1078	175.023	931.842	5.32	0.000510194
prc	EAMY_2023	211.297	1137.5	5.38	0.00100563
EAMY_3267	EAMY_3267	25.5776	138.425	5.41	7.52162E-05
EAMY_1768	EAMY_1768	159.37	872.813	5.48	0.0002551
flgH1	EAMY_1459	86.567	474.742	5.48	3.03253E-05
recG,spoU	EAMY_0047,EAMY_0048	122.041	676.004	5.54	2.41931E-06
araC	EAMY_1730	19.7405	112.409	5.69	4.39304E-05
ampC	EAMY_0516	82.7112	476.953	5.77	4.41885E-05
dgt	EAMY_2766	193.482	1127.24	5.83	0.000321751
pbpG	EAMY_1231	76.1751	444.02	5.83	2.13245E-05
vanA	EAMY_3549	24.2818	141.9	5.84	1.92679E-05
EAMY_3094,EAMY_3095	EAMY_3094,EAMY_3095	1355.51	7924.6	5.85	0.00434503
yfhC	EAMY_2612	209.614	1237.93	5.91	1.96123E-05
apaG,ksgA,pdxA,surA	EAMY_2924,EAMY_2925,EAMY_2926,EAMY_2927	661.99	3916.29	5.92	1.32448E-06
EAMY_0055	EAMY_0055	99.4406	590.17	5.93	7.10686E-05
trkA	EAMY_3353	105.809	636.956	6.02	7.57425E-05
rhtB3	EAMY_3256	171.333	1066.31	6.22	0.000018292
EAMY_1554	EAMY_1554	65.5806	410.47	6.26	0.00556557
EAMY_2274	EAMY_2274	3944.41	24706	6.26	0.000077798
araG	EAMY_1728	22.0742	138.656	6.28	0.000007644
EAMY_1750	EAMY_1750	52.1525	329.127	6.31	9.24007E-06
flgB1	EAMY_1453	54.1851	345.202	6.37	1.88064E-05
EAMY_0390,EAMY_0391, EAMY_0392	EAMY_0390,EAMY_0391,EAMY_0392	314.115	2003.23	6.38	6.42294E-06
EAMY_1756	EAMY_1756	794.307	5113.63	6.44	0.000615613
gltP	EAMY_3274	572.285	3697.09	6.46	0.000638476

EAMY_1697	EAMY_1697	123.026	797.527	6.48	5.05998E-05
yifB	EAMY_0157	143.052	927.714	6.49	9.82873E-05
yleB	EAMY_1137	173.07	1124.91	6.50	0.000081633
ydcR	EAMY_0480	142.527	945.606	6.63	7.77199E-05
yhhF,*yhhL*	EAMY_3497,EAMY_3498	499.793	3378.41	6.76	3.12239E-08
zntA	EAMY_3500	27.4261	185.494	6.76	6.01462E-06
EAMY_2140	EAMY_2140	56.4998	385.629	6.83	3.98253E-06
hrpK	EAMY_0519	84.5621	577.308	6.83	6.02393E-05
EAMY_2166	EAMY_2166	84.0178	575.952	6.86	0.00360302
EAMY_1658,*nlpC*	EAMY_1657,EAMY_1658	560.611	3888.38	6.94	0.000003116
EAMY_2455,EAMY_2456	EAMY_2455,EAMY_2456	281.928	1975.49	7.01	0.000403296
msuD	EAMY_1803	26.7904	189.64	7.08	2.08472E-06
fdhD	EAMY_3653	71.6579	518.388	7.23	2.65459E-06
EAMY_1598,EAMY_1599, *rtn2*	EAMY_1598,EAMY_1599,EAMY_1600	2056.03	15676.1	7.62	1.32932E-08
yrbF	EAMY_0323	333.158	2550.04	7.65	0.000029762
yhcF	EAMY_1861	140.184	1078.49	7.69	3.20449E-06
rtn3	EAMY_3517	183.285	1422.72	7.76	3.57817E-05
EAMY_3235	EAMY_3235	29.0628	225.672	7.76	1.14379E-06
ychJ	EAMY_1964	107.866	840.831	7.80	7.51548E-07
spaK	EAMY_0780	13.9364	109.035	7.82	0.000472924
pldB1	EAMY_0199	555.221	4353.48	7.84	3.03947E-05
lysR1	EAMY_0663	59.2583	468.788	7.91	1.01524E-06
EAMY_2963	EAMY_2963	156.15	1281.97	8.21	2.49681E-06
pmbA	EAMY_3127	120.57	1016.23	8.43	7.50679E-06
yehT,*yehU*	EAMY_0510,EAMY_0511	197.298	1678.93	8.51	2.02924E-07

cheB3,*cheR3*	EAMY_2696,EAMY_2697	137.944	1178.99	8.55	2.60731E-07
eamA	EAMY_2116	82.4874	713.612	8.65	7.79609E-07
ydaA	EAMY_1819	301.808	2613.25	8.66	1.26678E-05
ltaA	EAMY_1320	102.547	910.489	8.88	0.000001621
mmsA	EAMY_3514	417.456	3712.34	8.89	5.18791E-05
yhgH	EAMY_3452	64.8178	578.306	8.92	2.25213E-07
EAMY_1438	EAMY_1438	87.5702	783.006	8.94	0.00485546
ydgR	EAMY_1712	69.6298	623.605	8.96	1.8243E-06
EAMY_2015	EAMY_2015	2645.27	24051.7	9.09	1.53564E-07
ybjO	EAMY_1310	49.4025	474.805	9.61	7.94945E-08
EAMY_2044,*exoX*	EAMY_2044,EAMY_2045	505.975	4880.85	9.65	0.000002168
EAMY_0394	EAMY_0394	96.2871	1033.14	10.73	0.000049015
ydiJ	EAMY_1673	70.1636	807.594	11.51	8.66886E-07
proS	EAMY_2733	143.944	1730.73	12.02	6.42599E-07
proQ	EAMY_2024	348.185	4190.46	12.04	4.27199E-07
EAMY_1044	EAMY_1044	31.3569	380.066	12.12	6.17608E-09
EAMY_3550	EAMY_3550	212.69	2616.28	12.30	2.86402E-07
yveA	EAMY_2594	79.7088	1013.79	12.72	1.3416E-07
ygaD	EAMY_0804	83.5081	1102.46	13.20	2.39539E-09
baeR	EAMY_2266	82.6925	1105.26	13.37	1.07773E-08
yebH	EAMY_2014	294.883	4019.38	13.63	2.04286E-07
EAMY_1775	EAMY_1775	77.8278	1078.64	13.86	0.000673457
elaC	EAMY_3541	58.743	817.757	13.92	7.70212E-09
yhhN	EAMY_3499	188.296	2740.85	14.56	2.34973E-08
ybcQ1	EAMY_1517	51.373	792.379	15.42	3.31863E-10
EAMY_2584	EAMY_2584	197.938	3185.74	16.09	0.00000147

lrgA,ywgG	EAMY_3279,EAMY_3280	179.271	3178.73	17.73	4.19775E-12
EAMY_3111	EAMY_3111	170.848	3090.51	18.09	3.81579E-11
EAMY_0699	EAMY_0699	865.814	15947.8	18.42	2.86269E-08
EAMY_1266	EAMY_1266	1249.5	23067.7	18.46	2.00755E-11
EAMY_0503	EAMY_0503	101.177	1899.8	18.78	3.24066E-07
EAMY_2561	EAMY_2561	268.874	5048.66	18.78	0.000140905
yoaE	EAMY_2013	144.913	2737.77	18.89	5.16692E-09
EAMY_1620	EAMY_1620	57.3017	1096.59	19.14	0.000000001
gltI	EAMY_1130	61.5035	1200.09	19.51	5.5192E-10
EAMY_3230	EAMY_3230	413.908	8142.08	19.67	0.000109377
EAMY_0150	EAMY_0150	1373.94	27518.4	20.03	1.04849E-08
ampG	EAMY_0977	107.369	2161.83	20.13	1.47411E-09
fliE1	EAMY_1512	44.292	899.567	20.31	7.66605E-11
glpF	EAMY_0128	61.6822	1259.33	20.42	2.48888E-10
lgt,thyA	EAMY_0702,EAMY_0703	217.656	4575.96	21.02	4.59977E-11
ycfX	EAMY_1525	606.612	12820.4	21.13	4.59272E-10
rfbX,yibD	EAMY_2110,EAMY_2111	67.8039	1452.03	21.42	1.33227E-15
EAMY_1672	EAMY_1672	249.437	5352.79	21.46	0.00349748
EAMY_2337	EAMY_2337	182.322	3912.55	21.46	6.70616E-05
lysR3	EAMY_3561	89.3471	1954.87	21.88	2.18247E-10
proP3	EAMY_3688	15.824	359.384	22.71	2.23356E-09
EAMY_3459,*glpE*	EAMY_3458,EAMY_3459	1424.95	33378.6	23.42	2.22045E-15
EAMY_1239	EAMY_1239	161.247	3818.69	23.68	2.60633E-10
bglA	EAMY_0494	122.043	3114.76	25.52	1.02909E-10
yneJ	EAMY_2293	30.9294	793.424	25.65	2.37721E-12
EAMY_3289	EAMY_3289	453.906	12175.8	26.82	1.75917E-05

EAMY_1860	EAMY_1860	453.906	12987.5	28.61	1.16907E-05
fliZ	EAMY_2138	121.55	3950.98	32.50	2.40985E-12
iolC	EAMY_3510	43.2801	1414.57	32.68	2.65898E-12
yebU	EAMY_2028	62.7224	2065.27	32.93	2.4265E-12
EAMY_0998	EAMY_0998	315.445	13397.6	42.47	3.73535E-11
amiA	EAMY_2498	51.1934	2186.68	42.71	8.9706E-14
cybB	EAMY_3547	176.108	7620.16	43.27	7.79377E-14
gtrB3	EAMY_3311	65.014	3565.73	54.85	3.55271E-15
EAMY_1743	EAMY_1743	48.574	2719.37	55.98	2.22045E-15
EAMY_3163	EAMY_3163	114.547	6566.91	57.33	0.000101968
EAMY_1627	EAMY_1627	2276.49	141931	62.35	0
EAMY_2047	EAMY_2047	448.908	43299.6	96.46	0
EAMY_0794	EAMY_0794	116.974	17153.1	146.64	0
EAMY_0945,EAMY_0946	EAMY_0945,EAMY_0946	546.966	121859	222.79	0
EAMY_2636	EAMY_2636	274.787	91400.5	332.62	2.29358E-08
EAMY_2605	EAMY_2605	104.196	58260.2	559.14	0

Figure S3. Classes of *Erwinia amylovora* CFBP 1430 genes (GO terms) that were affected by the mutation of T6SS (comparing T6-d1d3 mutant to WT) representing the fire blight T6SS transcriptome. The significantly differentially expressed genes from the *in planta* transcriptomic data are depicted in the graph on top (A) and the *in vitro* data in the bottom graph (B).

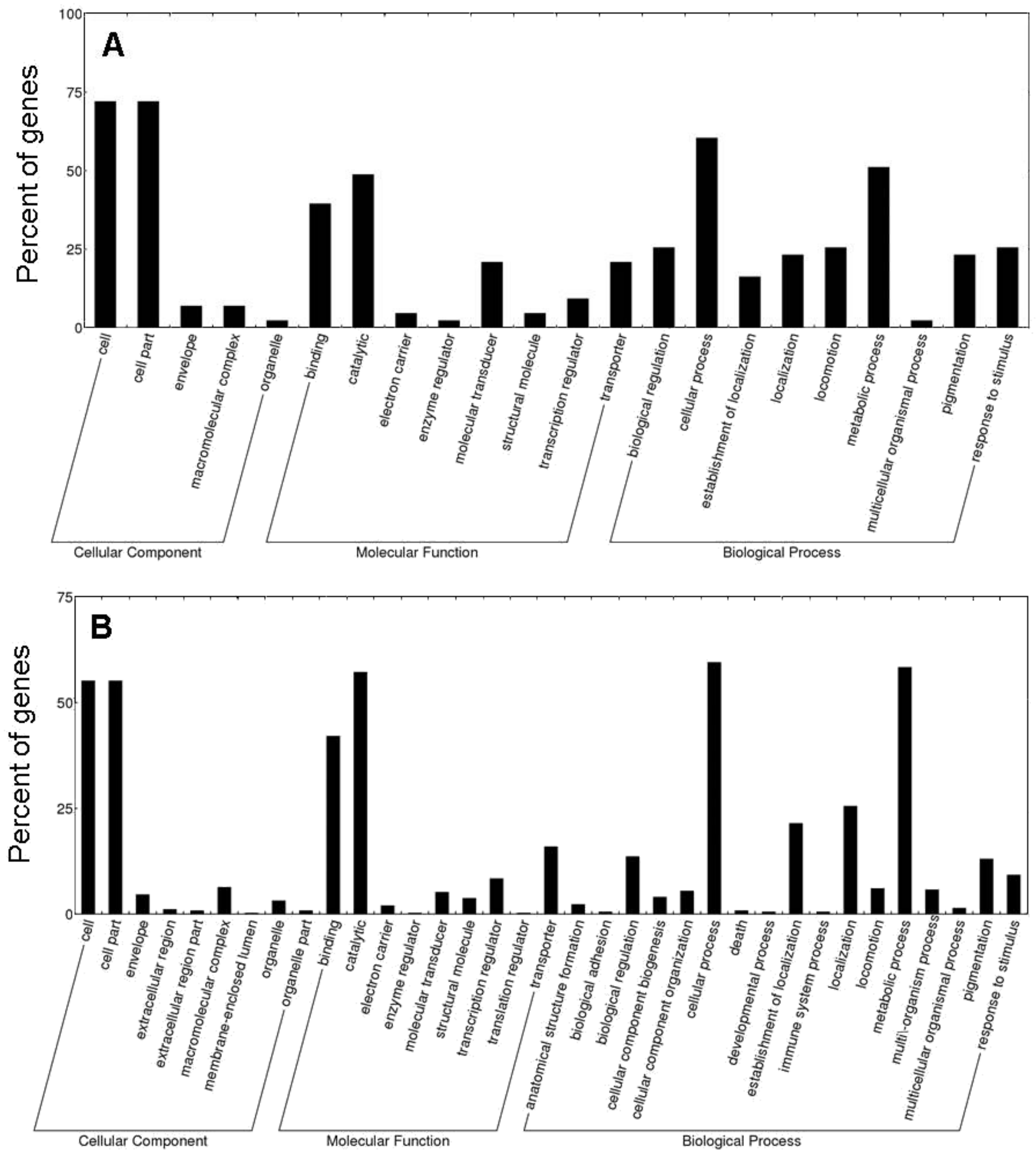

Figure S4. Immature pears were inoculated with water or a bacterial suspensions containing (**A**) 10^5 cfu ml^{-1} or (**B**) 10^7 cfu ml^{-1}. Pears were assessed 8 days post inoculation.

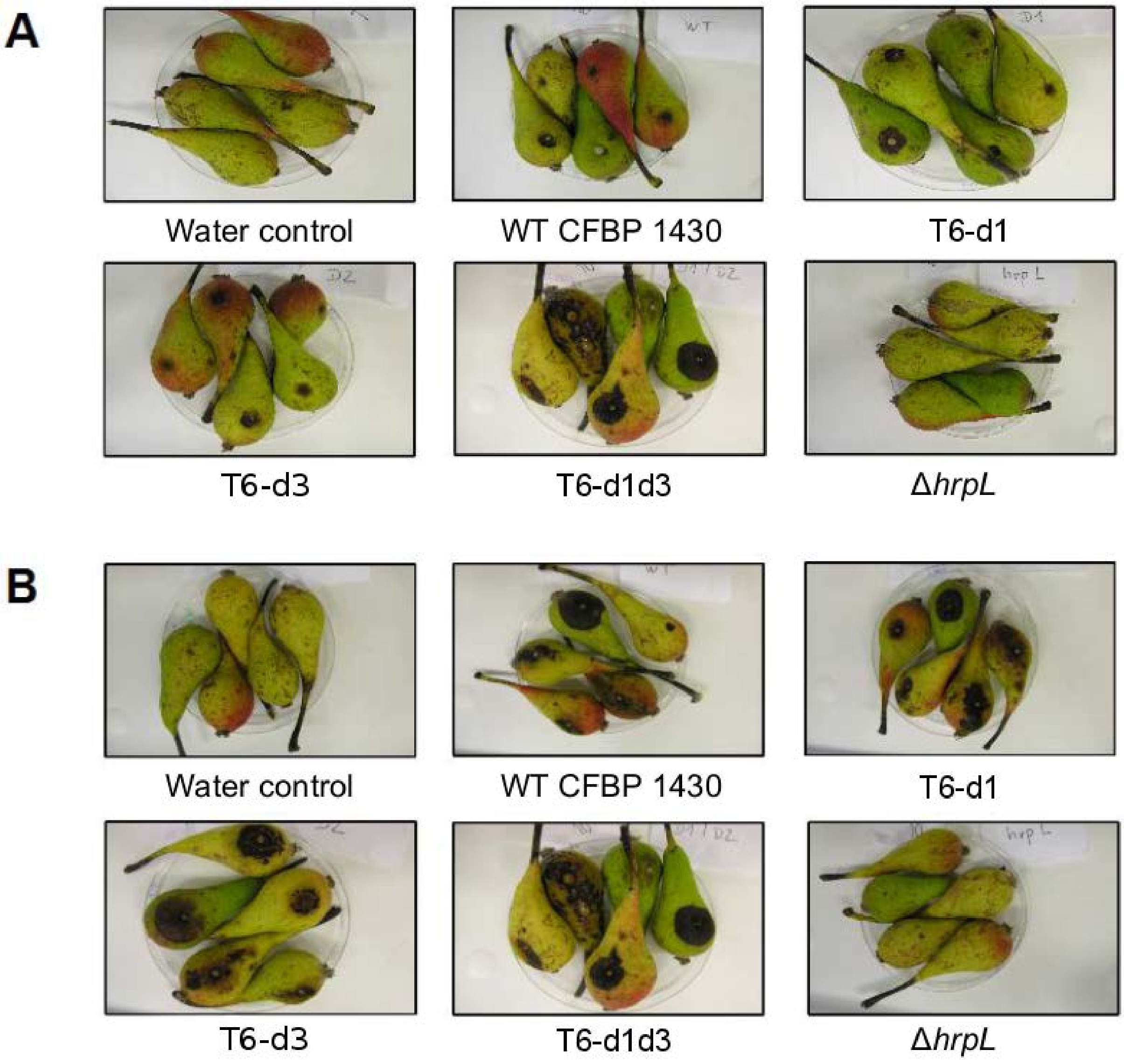

Figure S5. Bacteria were recovered from flowers at A) 1 DPI and B) 2 DPI. Colony forming units (cfu) were determined by dilution plating. An Anova was performed to test for statistical significant differences.

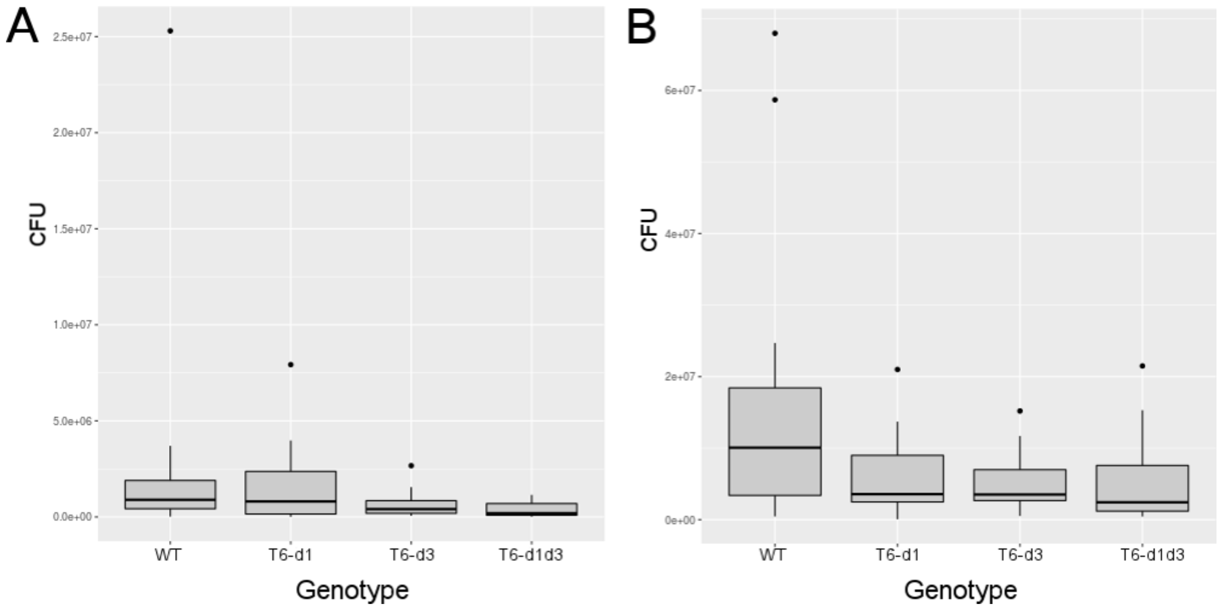

Table S3. Significantly differentially expressed genes from the in planta experiment

E. amylovora gene name	locus tag	FPKM(WT)	FPKM(T6-d1d3)	Fold change	P value
EAMY_3021	EAMY_3021	635.19	0	-0	2.96522e-17
EAMY_3020	EAMY_3020	478.65	4.54	-105.5059096551	0
EAMY_3529,EAMY_3530	EAMY_3529,EAMY_3530	1005.44	32.76	-30.6953879789	0.000505032
EAMY_2477	EAMY_2477	1947.51	817.01	-2.3836955766	2.9032e-08
pstA3,pstC3	EAMY_3692,EAMY_3693	194.25	81.68	-2.378133986	6.28619e-05
fliC1	EAMY_2141	34163.8	14815.7	-2.3059160649	4.65183e-13
pstS3	EAMY_3694	200.95	86.96	-2.3107320882	2.01134e-05
tauB,tauC,tauD3	EAMY_3404,EAMY_3405,EAMY_3406	439.05	203.7	-2.1553019503	4.58666e-07
EAMY_0934,EAMY_0935	EAMY_0934,EAMY_0935	1011.75	497.6	-2.0332533628	4.83212e-05
EAMY_3721*	EAMY_3721	1527.57	781.14	-1.955569758	1.78671e-07
tauA	EAMY_3407	218.86	112.01	-1.9539682159	0.000330024
phoH	EAMY_2126	341.19	178.29	-1.9136712365	0.00014718
cysC,cysN	EAMY_0754,EAMY_0755	923.96	491.96	-1.8781183792	2.91775e-09

cheY1	EAMY_2087	3922.69	2133.81	-1.8383481335	3.43631e-06
yfiA	EAMY_0830	2102.42	1150.27	-1.8277693181	3.26631e-05
metE	EAMY_0208	141.83	78.13	-1.8153516018	3.58437e-05
EAMY_1906	EAMY_1906	8573.45	4759.55	-1.801315636	3.02396e-05
EAMY_3565	EAMY_3565	180.53	103.04	-1.7520391344	0.000252358
yhhW	EAMY_3476	1026.29	585.1	-1.7540501543	2.21915e-05
EAMY_2140	EAMY_2140	885.74	507.09	-1.7467292475	2.13968e-05
pspA	EAMY_1877	1046.55	602.51	-1.7369822363	3.132e-05
flgL1	EAMY_1463	2512.86	1457.83	-1.7237075229	2.27793e-05
fabB	EAMY_2423	631.82	377.51	-1.6736316039	8.75327e-05
tsr7	EAMY_3131	1375.29	825.63	-1.6657535867	7.22886e-05
EAMY_0661	EAMY_0661	921.27	580.37	-1.5873893155	0.000639083
cheZ1	EAMY_2086	1933.15	1222.08	-1.5818480245	0.000464126
cheA1	EAMY_2095	1458.11	929.01	-1.5695347815	0.000418626
tsr3	EAMY_2093	809.88	516.16	-1.5690648711	0.000540276

cheB1,cheR1	EAMY_2088,EAMY_2089	1747.88	1129.48	-1.5475135053	3.54703e-06
ymcA,ymcB	EAMY_3333,EAMY_3334	538.45	354.19	-1.5202493788	0.000599853
cysI3,cysJ	EAMY_0747,EAMY_0748	661.48	450.46	-1.4684447647	4.80499e-05
motA1,motB1	EAMY_2096,EAMY_2097	3546.8	2476.83	-1.4319935555	9.33682e-05
cstA	EAMY_0513	204.87	336.25	1.6412670519	0.000207294
sdhA,sdhC,sdhD	EAMY_1164,EAMY_1165,EAMY_1166	732.29	1451.98	1.9828011491	2.05014e-05
rcsV	EAMY_2800	89.3	353.22	0.2528160569	1.42812e-06
ykfE	EAMY_0517	223.67	1496.95	6.6926612781	6.12843e-14
EAMY_3046,EAMY_3047	EAMY_3046,EAMY_3047	39.78	416.02	10.458035736	7.0631e-05
ampC	EAMY_0516	67.63	2132.44	31.5318956464	0
EAMY_3573,*kdpA*	EAMY_3572,EAMY_3573	10.21	2714.44	265.864989904	3.00631e-07
dgkA	EAMY_3328	0	144.3	0	3.16712e-05
EAMY_3521	EAMY_3521	0	40.78	0	0.000455559
yddG	EAMY_0506	0	38.16	0	1.10452e-05

*located on pEa29

Figure S6. Role of *E. amylovora* CFBP 1430 T6SS in bacterial competition. *E. amylovora* WT or one of the T6SS mutants was co-inoculated with *E. coli* DH5α at a ratio 1:1 on either KB (A) or LB (B) plates. The bacterial strains were recovered 24 h post inoculation and cfu were counted. Bars with standard deviation represent the means of three independent experiments. A one way ANOVA was applied with the Tukey test to identify statistical significant differences between treatments. On KB plates the *E. coli* DH5α showed a statistically significant higher survival rate when co-inoculated with T6SS double mutant.

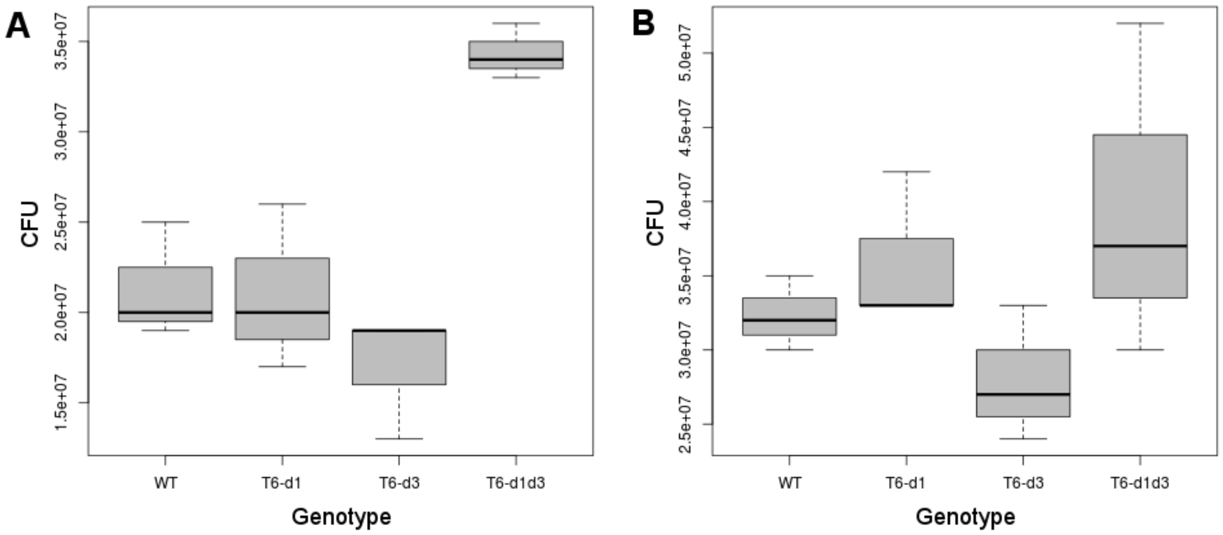

Supplement: Additional file 1: — This PDF file contains all supplementary tables and supplementary figures listed below. Table S1. Primers used in this study. Figure S1. Qualitative RT-PCR of selected T6SS genes. Figure S2. Growth curves with standard deviation measured for E. amylovora CFBP 1430 WT, T6-d1, T6-d3, and T6-d1d3 for 48 h in different media. Table S2. Significantly differentially expressed genes from the in vitro experiment. Figure S3. Classes of Erwinia amylovora CFBP 1430 genes (GO terms) that were affected by the mutation of T6SS (comparing T6-d1d3 mutant to WT) representing the fire blight T6SS transcriptome. Figure S4. Immature pears inoculated with E. amylovora CFBP 1430 or one of the T6SS mutants. Figure S5. E. amylovora CFBP 1430 and T6SS mutant populations on apple flowers at 1 DPI and 2 DPI. Table S3. Significantly differentially expressed genes from the in planta experiment. Figure S6. Role of E. amylovora CFBP 1430 T6SSs in bacterial competition. (PDF 633 kb) [file 12864_2017_4010_MOESM1_ESM.pdf]
